# Translation and Linguistic Validation of Outcome Instruments for Traumatic Brain Injury Research and Clinical Practice: A Step-by-Step Approach within the Observational CENTER-TBI Study

**DOI:** 10.3390/jcm10132863

**Published:** 2021-06-28

**Authors:** Nicole von Steinbuechel, Katrin Rauen, Ugne Krenz, Yi-Jhen Wu, Amra Covic, Anne Marie Plass, Katrin Cunitz, Isabelle Mueller, Fabian Bockhop, Suzanne Polinder, Lindsay Wilson, Ewout W. Steyerberg, Andrew I. R. Maas, David Menon, Marina Zeldovich, The Linguistic Validation Group of CENTER-TBI

**Affiliations:** 1 Institute of Medical Psychology and Medical Sociology, University Medical Center Göttingen, Waldweg 37A, 37073 Göttingen, Germany; ugne.krenz@med.uni-goettingen.de (U.K.); yi-jhen.wu@med.uni-goettingen.de (Y.-J.W.); amra.covic@med.uni-goettingen.de (A.C.); annemarie.plass@med.uni-goettingen.de (A.M.P.); katrin.cunitz@med.uni-goettingen.de (K.C.); isabelle.mueller@med.uni-goettingen.de (I.M.); fabian.bockhop@med.uni-goettingen.de (F.B.); marina.zeldovich@med.uni-goettingen.de (M.Z.); 2 Department of Geriatric Psychiatry, Psychiatric Hospital Zurich, University of Zurich, Minervastrasse 145, 8032 Zurich, Switzerland; katrin.rauen@uzh.ch or; 3 Institute for Stroke and Dementia Research (ISD), University Hospital, LMU Munich, Feodor-Lynen-Straße 17, 81377 Munich, Germany; 4 Department of Public Health, Erasmus MC, University Medical Center Rotterdam, 3000 CA Rotterdam, The Netherlands; s.polinder@erasmusmc.nl (S.P.); e.steyerberg@erasmusmc.nl (E.W.S.); 5 Department of Psychology, University of Stirling, Stirling FK9 4LJ, UK; l.wilson@stir.ac.uk; 6 Department of Biomedical Data Sciences, Leiden University Medical Center, 2333 RC Leiden, The Netherlands; 7 Department of Neurosurgery, Antwerp University Hospital and University of Antwerp, 2650 Edegem, Belgium; andrew.maas@uza.be; 8 Division of Anaesthesia, University of Cambridge, Addenbrooke’s Hospital, Box 157, Cambridge CB2 0QQ, UK; dkm13@cam.ac.uk

**Keywords:** translation, linguistic validation, outcome instruments, traumatic brain injury

## Abstract

Assessing outcomes in multinational studies on traumatic brain injury (TBI) poses major challenges and requires relevant instruments in languages other than English. Of the 19 outcome instruments selected for use in the observational Collaborative European NeuroTrauma Effectiveness Research in TBI (CENTER-TBI) study, 17 measures lacked translations in at least one target language. To fill this gap, we aimed to develop well-translated linguistically and psychometrically validated instruments. We performed translations and linguistic validations of patient-reported measures (PROMs), clinician-reported (ClinRO), and performance-based (PerfO) outcome instruments, using forward and backward translations, reconciliations, cognitive debriefings with up to 10 participants, iterative revisions, and international harmonization with input from over 150 international collaborators. In total, 237 translations and 211 linguistic validations were carried out in up to 20 languages. Translations were evaluated at the linguistic and cultural level by coding changes when the original versions are compared with subsequent translation steps, using the output of cognitive debriefings, and using comprehension rates. The average comprehension rate per instrument varied from 88% to 98%, indicating a good quality of the translations. These outcome instruments provide a solid basis for future TBI research and clinical practice and allow the aggregation and analysis of data across different countries and languages.

## 1. Introduction

Traumatic brain injury (TBI) is a major cause of lifelong disability worldwide [1]. It is defined as “an alteration in brain function, or other evidence of brain pathology, caused by an external force” [2] (p. 1637). A TBI may result in a variety of consequences, such as temporary or persisting functional disability [3]; neurological problems [4,5], including sensory-motor disorders [6,7], as well as neuropsychological [8,9], psychosocial, and psychiatric sequelae [10,11,12]; and reduced health-related quality of life (HRQOL) [13].

Given the broad range of areas affected, the complexity and heterogeneity of TBI and its consequences cannot be adequately captured by unidimensional outcome assessments [14]. The paradigm shift in classifying and treating TBI not only as an acute but rather as a chronic brain disease emphasizes the need for a multi-level outcome assessment [1,15,16], which should cover the various outcome domains and reflect the perspectives of both patients and healthcare professionals.

Over the past 35 years, outcome instruments have been developed for different clinical fields [17,18] and, during the last decade, TBI research has started to apply combinations of them [13,14,19]. Outcomes after TBI can be assessed using instruments based on clinician-reported outcomes (ClinROs), patient-reported outcomes measures (PROMs), and performance-based physical and cognitive outcomes (PerfOs). PROMs use patients’ self-ratings regarding their subjective perspective of their health condition and/or medical treatment [20,21]. In ClinRO instruments and clinical tests, the patients’ status is assessed by trained healthcare professionals, while PerfO instruments capture the “objective” functional performance through standardized tests, mostly carried out by psychologists or other clinical personnel [21].

Multicenter multinational studies that investigate outcomes multidimensionally by using these types of instruments are required to comprehensively characterize outcome and recovery trajectories after TBI. A prerequisite for reliable and valid national and international multidimensional investigations of outcomes after TBI is the availability of well-translated, linguistically validated, and internationally harmonized ClinRO, PerfO instruments, and PROMs to assess cognitive, psychological, and psychosocial outcomes, HRQOL, recovery, and amnesia in multiple languages. Many of these are, however, only available in a limited number of languages [22].

To overcome this limitation, the instruments need to be translated and linguistically validated in the target languages for international studies on TBI outcome. The linguistic validation of instruments is challenging as it needs to address the cultural and conceptual differences between the respective language while maintaining the contents of each instrument on a conceptual level across the different languages [23]. A systematic review found that no standardized international guidelines exist for the linguistic validation of health-related outcome instruments [24]. Nevertheless, several guidelines and recommendations for iterative translation procedures are in use, published by the MAPI Research Trust [25], the International Society for Pharmacoeconomics and Outcomes Research (ISPOR) [26,27], and others [23,28,29]. Moreover, further research is addressing the issue of the cross-linguistic adaptation of PROMs [26], ClinRO and PerfO instruments [30], and clinical ratings (e.g., Reference [31]). To date, general principles include the following steps:

First, the team coordinating the translation of an instrument should identify and clarify the concepts behind the instructions, items, and response formats (together with the developer) [25]. The translation of the original instrument into the target language should be performed by two independent native speakers, living in the country, fluent in English, briefed concerning the translation of health-related outcome instruments, and ideally having already performed this kind of translation before [25,26]. Second, the two translations should be combined to form a single forward version [25,28]. This reconciled version is back-translated by one independent linguist—a native speaker in the language of the original instrument and fluent in the target language—living in the respective country [24]. The reconciled target version is then revised considering the backward translation. Third, the target version should be cognitively debriefed in five to ten patients [25]. The amendments suggested by the target language translators are reviewed by the language translation coordinating team, discussed with the team and the target language translators (and the developer); then the translated instrument is finalized [25]. Finally, if an instrument is simultaneously translated into several languages, these translations should be internationally harmonized to ensure they are comparable, a process that is performed in the translation coordinating center together with the instrument’s authors [24]. These steps are meant to ensure that an instrument translation is “conceptually and linguistically equivalent to the source measure and allows data pooling and analysis/comparison across countries” [25] (p. 21).

While designing the Collaborative European NeuroTrauma Effectiveness Research study (CENTER-TBI; EC grant 602150; clinicaltrials.gov NCT02210221), a large international observational European study on TBI, we found that 17 of the 19 selected instruments or subtests were not available in at least one target language. To deal with this challenge, we decided to conduct translations and linguistic validations of these outcome instruments into up to 20 target languages, following most of the recommendations mentioned above. These translated instruments have been made available to the international scientific and clinical community. The present study describes the first part of the linguistic validation process of the outcome instruments administered in the CENTER-TBI study. The second part, concerning the psychometric properties of the PROMs is also published in the same issue of this journal [32].

## 2. Methods

### 2.1. Languages

The CENTER-TBI study was conducted from 2015 to 2017, across 18 countries in Europe and in Israel. The study protocol has been published and descriptive results have been presented [33,34]. The target languages for the linguistic validation were determined by the language(s) spoken in those countries that had expressed an interest in participating (Table 1).

### 2.2. Instruments

The selection of the outcome instruments was informed by the Common Data Elements (CDE) recommendations [35,36] taking into consideration TBI specificity and the free availability of instruments. As a result, the following outcome instruments were administered in the CENTER-TBI study (see Table 2). For a detailed description, see Appendix B.

### 2.3. Translation and Linguistic Validation Procedure

Outcome instruments were identified for which published translations and linguistic validations were not available in the languages required for the countries participating in the CENTER-TBI study. For these instruments, translations and linguistic validations were performed between October 2013 and October 2015. Table 3 gives an overview of the pre-existing translations and the translated and linguistically validated versions in the target languages of the participating countries.

The translations and linguistic validations of all instruments were coordinated by the core team of the University Medical Center Göttingen (UMG), consisting of one project coordinator and seven language coordinators, who led one to three translation teams in the participating countries. The core team included individuals whose language proficiency covered all required languages. In addition, the core team included at least one native speaker of each target language who was also fluent in English and who was responsible for that language. The core team and the translation teams in the participating countries comprised physicians, psychologists, teachers, linguists, nurses, occupational therapists, certified translators, administrative personnel, teachers, etc., who were experienced either in TBI research or clinical practice, outcome measurements, and/or translation.

We used a linguistic validation procedure that was guided by the recommendations of the MAPI Research Trust [24,25], adapted to the conditions of CENTER-TBI. As for various reasons (e.g., some centers dropped out of the study), some translations could not follow the entire process, we distinguish between *translation*, *linguistic validation* (without cognitive debriefings), and *full linguistic validation* (including cognitive debriefings). The outcome instruments were divided into two groups: (1) the core instruments (GOSE, QOLIBRI-OS, and SF-12v2) complemented by the additionally prioritized instruments (i.e., GOSE-Q, PCL-5, and QOLIBRI) and (2) the other instruments (GOAT, GAD-7, PHQ-9, RPQ, SF-36v2, RAVLT, TMT-A, B, and CANTAB). The procedure differed slightly for the two groups (see Phase 2).

#### 2.3.1. Phase 1—Conceptual Analysis of the Original Instrument

A concept list was devised as a basis for the translations, enumerating difficulties encountered during the prescreening of the instruments, to ensure that every translator was familiar with the constructs used in the instruments (i.e., for the GOSE, GOSE-Q, GOAT, PCL-5, RPQ, QOLIBRI/QOLIBRI-OS, and RAVLT). This list included explanations concerning the translation of English idioms, words with multiple meanings, symptoms, and their intensity. For example, the item “As a result of your injury are there now problems in how you get on with friends or relatives?” from the GOSE-Q was explained by noting that in this context “get on” means “get along with”. The item “Being ‘superalert’ or watchful or on guard” from the PCL-5 was explained as meaning very or extremely attentive. Moreover, translators were instructed to translate the Likert response scales considering the hierarchical order of the answers (e.g., from “not at all” to “extremely”) and their equidistance, etc. Concerning the PerfOs, some of the examples are presented here. For the RAVLT, translators were encouraged to use culturally adapted translations for the word “church”. Furthermore, this memory test includes homonyms, for which explanations were given, e.g., earth—the planet; turkey—the animal; orange—the fruit, etc. To further facilitate the comparability of the different RAVLT language versions, translators were asked to use a frequency list of words for each language (e.g., http://corpus.rae.es/lfrecuencias.html (accessed on 16 April 2021) for Spanish) to ensure that words with comparable frequencies were used. These strategies were adopted to support the comparability of the different language versions.

The explanatory concept list was discussed with all translation teams in the target languages/countries. In case of conceptual problems, the authors of the instruments were contacted.

#### 2.3.2. Phase 2—Translation: Forward Translations, Reconciled Version, and Backward Translation

All the original instruments were available at least in English. For the GAD-7 and PHQ-9, translations and validations had already been published in (most of) the target languages and were freely available (https://www.phqscreeners.com (accessed on 16 April 2021)). Licenses for the use of the SF-36v2, SF-12v2 were obtained from Optum [52]. Thus, the GAD-7, PHQ-9, and SF-36v2/-12v2 were administered as such. For the GOSE, GOSE-Q, PCL-5, QOLIBRI, and QOLIBRI-OS, two independent forward translations were performed into several target languages by native speakers. The respective translations were reconciled into one version by the translation team in the target language. These reconciled versions were then revised and adapted by the core team, in agreement with the target language translation team. A native English speaker who was not familiar with the original English instrument translated the harmonized forward translation back into English.

**Figure 1:**
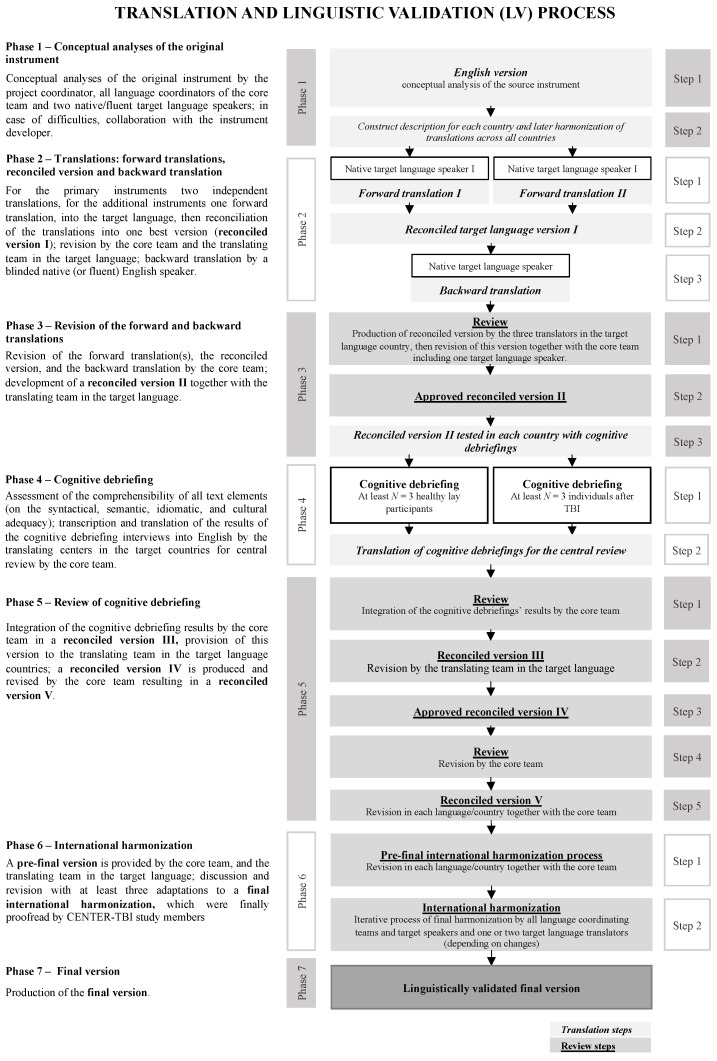
Translation and linguistic validation process.

All other instruments (i.e., GAD-7, PHQ-9, RPQ, RAVLT, TMT-A,B, and CANTAB subtests) underwent a single forward translation, due to limited resources. The test materials for the PerfO instruments comprise examples, visual and auditory materials which were—where appropriate—also subjected to the translation procedure. The other steps described above were the same for all instruments. See Figure 1 for an overview.

#### 2.3.3. Phase 3—Revision of the Forward and Backward Translations

A review of the original instrument, the forward translation(s), the reconciled version, and the backward translation was carried out by the core team. Reconciled versions were then agreed upon together with the translation teams in the target language countries.

#### 2.3.4. Phase 4—Cognitive Debriefing

These interviews, referred to as cognitive debriefings, are based on detailed structured questions whereby all answers are recorded. In the structured interview, participants were asked to share their thoughts about the meaning of each word, phrase, and item, and to comment on their comprehension of the respective instrument. The goal of a cognitive debriefing is to determine whether participants understand the text in the same way as it is intended in the original version of the instrument and whether its translation is culturally appropriate. These cognitive debriefings were performed in three to five individuals after TBI. Before this, three to five healthy individuals participated in the cognitive debriefings to anticipate and modify possible semantic, syntactical, idiomatic/pragmatic, and cultural issues early in the process. Clinicians were interviewed for the ClinROs.

The results were transcribed and translated into English by the translation teams. When linguistic and cultural problems were identified, the translations were further modified. The GOSE, the GOSE-Q, PCL-5, QOLIBRI, and QOLIBRI-OS underwent cognitive debriefings, which resulted in a *full linguistic validation*. No cognitive debriefings were performed for the other instruments (*linguistic validation*).

#### 2.3.5. Phase 5—Review of Cognitive Debriefing

The results of the cognitive debriefings were reviewed in the target languages by the core and the translation teams: if there were linguistic and/or cultural issues, alternative wording suggested by the lay people and patients interviewed (or clinicians, in case of clinical ratings) was integrated into an updated version of the instrument.

#### 2.3.6. Phase 6—International Harmonization

Final harmonization was performed by the core team for all the instruments. Furthermore, telephone or video conferences were held with the target language translation centers. In these, all concepts, such as cultural and linguistic equivalence, and all formal aspects were again discussed in detail, and if necessary appropriate adjustments were made. These versions were proofread by informed native speakers (investigators, participants, and management committee [MC] members of the CENTER-TBI study) for final adjustments.

#### 2.3.7. Step 7—Final Version

Based on the revisions and results of the international harmonization, a final version of each of the instruments was produced.

### 2.4. Evaluation of Translations

The comparability of the translations was assessed by numerically coding any semantic, cultural, idiomatic/pragmatic, and syntactic/grammatical differences, first comparing the original instrument version with the first harmonized version and then comparing this with the internationally harmonized final version. This coding procedure was designed to examine whether the translations captured the original instruments as closely as possible. The semantic level included all changes and problems related to the meaning of words and use of vocabulary. Cultural differences reflected the cultural relevance of the translations in the respective target languages. Idiomatic/pragmatic issues dealt with the translation of English idioms into the target language, for example. Finally, the syntactic/ grammatical level included, e.g., sentence structure, punctuation, etc. To quantify the changes, we assessed the differences in the instructions, items, and response categories of the instruments at these four levels. The number of differences is expressed as a percentage (i.e., number of differences relative to the total number of text elements in question). This number varies from 0% (no differences at all) to a maximum of 900% (multiple differences). Values above 100% indicate multiple modifications in one text element (e.g., nine coded differences in one instruction are expressed as 900%). The same modification in the same item text is considered once (e.g., the use of the courtesy pronoun in ten items of an instrument is counted as one modification). To summarize the results, we have provided an average percentage of changes across the languages for each instrument. To avoid the influence of outliers (e.g., extensive number of changes in a few languages), mean and median percentages of coded differences are reported.

Additionally, we have reported issues identified in the cognitive debriefings using comprehension rates. These were calculated by taking the number of individuals who participated in the cognitive debriefings, who had no problems understanding the instructions, items, and responses, and who had no concerns regarding the phrasing and the cultural conformance, and dividing this number by the overall number of interviewees. Mean comprehension rates were evaluated using quartiles, a commonly used measure in health sciences providing information about the center and the spread of the data. A rate of 100% indicates full comprehension, values above 75% were considered good, values ranging from 25% to 75% acceptable, and values below 25% indicated poor understanding. The results of the cognitive debriefings and reports on the translational and linguistic validation issues and solutions informed further revisions and harmonization of the instruments.

To assess these issues, the following questions were asked about the instructions, each item, and the respective response categories:

1. Did you have difficulties understanding this instruction/the question/the response options?
2. What did you understand this to mean?
3. Is it relevant for your situation?
4. Are the response options clear and consistent with the question?
5. If anything was misleading or unclear, how would you reword it?

## 3. Results

In total, 237 translations and 211 linguistic validations were carried out in up to 20 languages, including 14 translations and 12 linguistic validations of one ClinRO, 20 translations and 18 linguistic validations of its questionnaire version, 20 translations and 18 linguistic validations of the clinical amnesia test, 63 translations and 55 linguistic validations of the six PRO instruments, and 120 translations and 108 linguistic validations of the PerfO instruments (see Table 4).

### 3.1. Forward and Backward Translations, Comparison between the Original Version and the First Harmonization (Phases 2 and 3)

The forward and backward translations were performed for all outcome instruments. Some GOSE-Q translations conducted for the European multi-center Eurotherm study [53] were used as the forward translation (Dutch, German, French, Hungarian, Italian, Lithuanian, Russian, and Spanish). All instruments were then back-translated. Already existing and published translations of the Dutch and French GOSE, and the French translations of the QOLIBRI/QOLIBRI-OS were edited according to the comments of the translators and revised in an iterative process during the international harmonization (Phase 6).

The comparison between the original English versions and the first harmonization mainly revealed differences at the semantic level, followed by idiomatic and cultural issues (see Table 5). Considering both the mean and median percentage of differences, most changes were observed for the RPQ, followed by the GOSE-Q, GOSE, PCL-5, and the RAVLT.

At *the semantic level*, specifically, the term “(head, brain) injury” underwent semantic changes across many languages. Frequently, “injury” was translated as “trauma” which seemed more appropriate in the respective language contexts. Further semantic changes concerned the choice of words, with the aim of capturing the original instruments as closely as possible.

At the *idiomatic/pragmatic level*, the differences between translations comprised adaptations of English idioms and special phrases. For example, the question “How are you satisfied with your ability to get out and about” from the QOLIBRI had to be explained, as the idiom “get out and about” is phrased differently in many languages.

The *cultural level* included the use of specific pronominal forms, gender-appropriate language, and the translation of specific terms lacking or seldom used in the culture of the target language. Here, two tendencies were observed: (1) more informal gender-neutral translations (especially in Northern European languages) and (2) more formal gender-sensitive translations in other European languages and Hebrew. In addition, as already expected after devising the conceptual list, differences occurred in the translation of the explanatory text of the GOSE-Q, which included an example of “playing bingo” among other leisure activities. Since playing bingo is uncommon as a leisure activity in many countries, many translating teams used a culturally more appropriate example such as “going out to a restaurant”. For a detailed overview, see Supplementary Materials Table S1 online.

### 3.2. Cognitive Debriefings and Quality of Translations (Phases 4 and 5)

Overall, the average comprehension rates of the participants interviewed for each instrument were above 90% for items and response categories and greater than or equal to 85% for the instructions (see Table 6). Some translations with lower comprehension rates (e.g., the Swedish GOSE/GOSE-Q, the instructions of the Slovakian GOSE-Q, the instructions and responses of the French GOSE-Q, the instructions of the French PCL-5, and the responses of the Slovakian QOLIBRI) required further revisions, which were carried out in the next harmonization step. However, all comprehension rates were at least within an acceptable range (25% to 75%) or above (≥75%), except for the instructions of the Swedish version of the GOSE and the French version of the GOSE-Q, where all participants commented on the wording, which was corrected.

The translational challenges determined in the cognitive debriefings for the ClinRO, its questionnaire version, the PROMs on a linguistic (semantic, syntactic, and idiomatic), and cultural level are summarized in Table 7, together with their solutions.

Linguistic and cultural differences can occur not only in the translation of PROMs but also in the translation of PerfO instruments. The RAVLT can serve as an example of the complexity of the translational and linguistic validation process. A good example of some cultural differences is the word “church”, which is used less often in countries where religious backgrounds other than Christianity are predominant. As a solution, the use of the terms “mosque”, “synagogue”, “temple”, or “church” was implemented for these translations. It was also noticed that one to two-syllable nouns were usually used in the English version of the RAVLT, which is not the case in all languages. In the Lithuanian, Russian, and Hungarian languages, for example, nouns generally have two or more syllables, as reflected by the translated nouns. Since the number of syllables per word may influence verbal memory, such language-specific characteristics need to be considered for further multinational translation procedures concerning verbal memory.

### 3.3. Harmonization and Final Versions (Phases 6 and 7)

The harmonized versions underwent further revisions, depending on the complexity and conceptual clarity of the instrument, the quality of the translations, and results of the cognitive debriefings. When different opinions arose among the team members involved in the final national and international harmonization, a consensus was sought resulting in the most appropriate translations. Instrument developers were only contacted when problems could not be solved, which only happened twice (for the GOAT and PCL-5). These versions were reviewed by informed native speakers (members of the CENTER-TBI study) in the different languages for final adjustments.

Table 8 provides an overview of the changes in coding the semantic, cultural, idiomatic/pragmatic, and syntactic/grammatical differences and issues between the first harmonization and the final versions administered in the study. Most differences concerned semantic and syntactic/grammatical changes. The changes contained improvements of inappropriate translations, consistent use of gender-appropriate language, and grammatical issues (e.g., use of commas and spelling). Most of the issues involved the use of synonyms or words that were initially translated literally from English into the target language but that were not suitable in the context of this language.

Considering the average (i.e., the mean) relative number of coded differences, the instruments involving the most changes were the RPQ, followed by the GOSE-Q and the PCL-5. However, the main changes in the RPQ, and in the PCL-5 occurred only in a few languages (Swedish, German, Danish, and Bosnian/Croatian/Serbian), which is reflected in the average percentages. Based on the median, most changes were performed during the harmonization of the GOSE-Q, followed by the RAVLT and the GOSE. For a detailed overview, see Supplementary Materials Table S2 online.

In sum, these efforts resulted in linguistically validated translations into up to 20 languages of one ClinRO instrument, its questionnaire version, one clinical amnesia test, six PROMs, and three PerfO instruments, including six CANTAB subtests. 

## 4. Discussion

The translations of the 17 outcome measures into up to 20 languages and the linguistic validations in up to 18 languages were achieved with input from over 150 international collaborators. These translated instruments provide a basis for reliable and valid future TBI outcome research and clinical practice, thereby facilitating data collection and comparisons across 18 countries in Europe and Israel. Translated and linguistically validated versions of the instruments used in the study are accessible in the public domain on the website of CENTER-TBI (https://www.center-tbi.eu/ (accessed on 16 April 2021)).

*Recommendations*: The following recommendations resulting from our linguistic validation work may be helpful for future international projects in the field of TBI and related areas.

*First*, it is important to work with translators with linguistic expertise, as well as expertise in the field of TBI, in addition to being native or fluent in English. In contrast to the MAPI guidelines [25], we only seldom resorted to certified professional translators as the professional translations had to be revised much more extensively in our study than the others. Furthermore, similar to Swaine-Verdier et al. (2004) [28], we also attempted—whenever possible—to integrate at least one person with a background in linguistics, teaching, or administration in the translation process. These vocational groups were assumed to be especially sensitized to the everyday use of language, comparable to the language of the individuals later answering the instruments [28]. In addition, regardless of the type of outcome measure (PROM, ClinRO, clinical amnesia test, or PerfO), it is important to consider the linguistic concepts and cultural background of each country, including the use of gender-appropriate language, courtesy, or informal pronominal forms, and specific idiomatic and pragmatic terms. Instruments with items using different and/or item-specific response formats and more detailed instructions do require more work compared with instruments consisting only of a few items with a standard Likert response scale. The translation and linguistic validation of clinical rating scales, such as the GOSE, were particularly time-consuming and complex. Detailed standardized training of raters is also recommended for this type of clinical scale, to enhance the comparability of the ratings.

*Second*, besides the selection of translators, the integration of extensive international harmonization panels and the good to excellent psychometric properties of the translated and linguistically validated PROMs described in von Steinbuechel et al. [32], as well as in van Praag et al. [54] and in Plass et al. [55], underline the quality of our translations of the instruments based on the procedure applied. Furthermore, we found face-to-face or video conferences very helpful during this procedure to enhance coherence across all languages. Visiting some translating country centers that participated in the linguistic validation procedure in person also served to intensify and ameliorate the process.

*Third*, as an understanding of the importance, implications, and intense workload of linguistic validation procedures is still underrepresented in the field of TBI research, more resources should be allocated to this type of undertaking. For future international multidimensional outcome studies on TBI, we wish to provide a solid basis for linguistic validation and its funding.

*Fourth*, the issue of commercial ownership of instruments is one that public funding bodies such as the EU and the National Institutes of Health (NIH) should consider before designating instruments as recommended data elements. While the costs involved in using such commercial instruments may not be an insurmountable burden in high-income countries, they may be extremely challenging in low- and middle-income countries. The SF-12v2, SF-36v2, and the CANTAB are only available on a commercial basis. All translations of the QOLBRI and the QOLIBRI-OS instruments described in this manuscript are freely available for academic use from www.center-tbi.eu (accessed on 16 April 2021) and https://qolibrinet.com (accessed on 16 April 2021). In addition, translations of these instruments developed within the CENTER-TBI project (Arabic, Bulgarian, Bosnian/Croatian/Serbian, Hebrew, Hungarian, Latvian, Lithuanian, Romanian, Russian, Slovakian, and Spanish) are also free for commercial use. All other translations of these instruments that preceded the CENTER-TBI project are not covered by CENTER-TBI agreements; they are free for academic research, but not for commercial use. For further information and potential (commercial) use, please access https://qolibrinet.com (accessed on 16 April 2021).

*Fifth*, many of the outcome instruments are available in a wide range of versions, the existence of which is not obvious. Resources, therefore, need to be used to identify appropriate versions ensuring the comparability of data.

*Sixth and final*, as the translation and linguistic validation procedures were labor- and resource-intensive, they could only be accomplished thanks to the dedication and using the personal resources of contributing CENTER-TBI participants, investigators, and MC members.

Overall, the freely available ClinRO, its questionnaire version, a clinical amnesia test, PROMs, and PerfOs and the results of the present study provide many opportunities for future translations and linguistic validations in other languages. The psychometric validation of the PROMs [32] in different languages establishes a reliable basis for national, international, and multicenter studies in the field of TBI.

*Limitations*. The translation and linguistic validation procedure described in the present study mainly followed the recommendations of MAPI [25] and ISPOR [26,27], with some adaptations in terms of the conceptual analyses, selection of translators, and the number of translations and cognitive debriefings. In contrast to these recommendations, we only contacted developers for the conceptual analysis of the original instruments when difficulties occurred with the concept description or if difficulties arose during the harmonization. Translators did not need to provide a language certificate but needed to be native speakers, fluent in English, experienced in the care of patients in the field of TBI, and preferably in outcome assessment. The effectiveness of this type of selection of professionals was also reflected in the good to excellent psychometric quality of the translations [53,54,55]. As the labor-intensive procedures required a pragmatic approach with an efficient use of limited resources, only one formal forward translation was carried out for the non-core or not additionally prioritized instruments (i.e., GOAT, GAD-7, PHQ-9, RPQ, TMT-A, B, RAVLT, and CANTAB).

However, we tried to compensate for potential deficits by means of intense iterative reviews, revisions, and international harmonization. Concerning the cognitive debriefings performed, we interviewed at least two clinicians, three-to-five laypersons, and three to five TBI patients, instead of the minimum five described by MAPI [25], which was an effective way of coping with the shortage in some countries (centers) of TBI patients willing to participate in the cognitive debriefings.

Finally, we decided to delay publication until the data and results of psychometric analyses of the translated instruments were available [32], which required completion of enrolment and six-month outcome assessment (2018) and subsequent data curation and analysis (2020 to 2021).

*Future perspectives.* Future research should perform cognitive debriefings and international harmonization of the Arabic, Russian, Bulgarian, and Czech translations, which have not been carried out. In the next step, the psychometric characteristics and measurement invariance of the concepts used in the different instruments should be a topic for future research [56,57,58].

## 5. Conclusions

Linguistically validated translations in up to 20 languages were produced for one ClinRO (GOSE) and its questionnaire version (GOSE-Q), one clinical amnesia test (GOAT), six PROMs (GAD-7, PHQ-9, PCL-5, RPQ, QOLIBRI, QOLIBRI-OS), and three PerfO instruments (RAVLT, TMT-A,B) and the CANTAB with six separate subtests for individuals after TBI. The translations of the outcome instruments, in so far as they are in the public domain, are available on the CENTER-TBI homepage (https://www.center-tbi.eu/, accessed on 16 April 2021).

The description of the linguistic validation process of these instruments may provide the basis for future linguistic and psychometric validations for national and multinational cross-cultural TBI outcome studies. The availability of these instruments with good to excellent psychometric properties in the field of TBI [32] in the most widely spoken languages in Europe and Israel may facilitate and improve outcome research and clinical evaluation of individuals after TBI in the future.

## Figures and Tables

**Table 1:** Target languages for the linguistic validation of the countries that participated in the CENTER-TBI study.

No.	Target Languages	Country
1	Arabic	Israel
2	Bosnian/Croatian/Serbian	Bosnia/Croatia/Serbia
3	Bulgarian	Bulgaria
4	Czech	Czech Republic
5	Danish	Denmark
6	Dutch	Belgium, the Netherlands
7	Finnish	Finland
8	French	Belgium, France
9	German	Austria, Belgium, Germany
10	Hebrew	Israel
11	Hungarian	Hungary
12	Italian	Italy
13	Latvian	Latvia
14	Lithuanian	Lithuania
15	Norwegian	Norway
16	Romanian	Romania
17	Russian	Israel
18	Slovakian	Slovakia
19	Spanish	Spain
20	Swedish	Sweden

Note. As the Bulgarian and Czech centers dropped out of the CENTER-TBI study early on, not all linguistic validation steps could be performed (see results and discussion). Moreover, cognitive debriefings were not carried out for the Arabic and Russian language. Thus, these languages are not available on the CENTER-TBI website, but from nvsteinbuechel@med.uni-goettingen.de for further linguistic validation.

**Table 2:** Outcome instruments administered in the CENTER-TBI study.

Abbreviation	Instrument	Outcome Domain	No. of Items	Response Format	Response Categories
**clinician-reported outcome instrument (ClinRO), its questionnaire version, and a clinical amnesia test**					
GOSE*	Glasgow Outcome Scale Extended [37]	Functional outcome and level of disability	19	Dichotomous and polytomous	“yes”/“no” (**16 items**) and item-specific rating scales and response categories (**three items**)
GOSE-Q	Glasgow Outcome Scale Extended—Questionnaire version [38]	Functional outcome and level of disability	14	Dichotomous and polytomous	“yes”/“no” (**five items**) and item-specific rating scales and response categories (**nine items**)
GOAT	Galveston Orientation Amnesia Test [39]	Post-traumatic amnesia	10 and 3 sub-items	Dichotomous	Error evaluation by clinician
**Patient-reported outcome measures (PROMs**)					
GAD-7	Generalized Anxiety Disorder 7 item scale [40]	Psychological outcome (generalized anxiety disorder)	7	Polytomous	“not at all” “several days” “more than half the days” “nearly every day”
PHQ-9	Patient Health Questionnaire 9 [41]	Psychological outcome (depression)	9 items and 1 additional question	Polytomous	“not at all” “several days” “more than half the days” “nearly every day” (**nine items**) **and** “not difficult at all” “somewhat difficult” “very difficult” “extremely difficult” (**one item**)
PCL-5	Posttraumatic Stress Disorder Checklist [42]	Psychological outcome (post-traumatic stress disorder)	20 items and 1 additional question	Polytomous and one dichotomous item	“not at all” “a little bit” “moderately” “quite a bit” “extremely” **and** “yes”/“no” (**one item**)
RPQ	Rivermead Post-concussion Symptoms Questionnaire [43]	Psychological, cognitive, and behavioral outcome	16 and 1 additional question	Polytomous and two semi-open questions	“no more of a problem” “a mild problem” “a moderate problem” “a severe problem” **and** the possibility of listing two further difficulties and rating them on the same scale
QOLIBRI	Quality of Life after Brain Injury Scale [44,45]	TBI-specific HRQOL	37 items	Polytomous	“not at all” “slightly” “moderately” “quite” “very”
QOLIBRI-OS *	Quality of Life after Brain Injury—Overall Scale [46]	TBI-specific HRQOL	6 items	Polytomous	“not at all” “slightly” “moderately” “quite” “very”
SF-36v2	Short Form Health Survey—Version 2 [47]	Generic HRQOL	36 items	Polytomous	Different kinds of Likert scales and item-related rating scales and response categories
SF-12v2 *	12-Item Short Form Survey—Version 2 [48]	Generic HRQOL	12 items	Polytomous	Different kinds of Likert scales and item-related rating scales and response categories
**Performance-based outcomes (PerfO)**					
CANTAB	Cambridge Neuropsychological Test Automated Battery ** [49]	Neuropsychological outcome	6 subtests (RTI, SWM, PAL, RVP, AST, SOC)	-	-
RAVLT	Rey Auditory Verbal Learning Test [50]	Neuropsychological outcome	-	-	Three versions of two respective word lists, 15 words each (A and B, four versions of the instrument for repeated testing)
TMT-A, B	Trail-Making Test A, B [51]	Neuropsychological outcome	-	-	TMT-A: numbers from 1 to 25 TMT-B: letters (A–L) and numbers (1–13)

* Instruments marked with an asterisk were selected as core instruments of the CENTER-TBI study [33]. ** For the computer-based CANTAB tests, the instructions and procedure descriptions were subjected to translation. The responses are language free as the test battery consists of visual and auditory stimuli to which subjects react on the behavioral level: RTI = reaction time, SWM = spatial working memory, PAL = paired associate learning, RVP = rapid visual processing, AST = attention switching task, SOC = stockings of Cambridge. HRQOL = health-related quality of life. Bold was used to highlight the number of items in the questionnaire.

**Table 3:** Pre-existing translations and translated and linguistically validated versions in the target languages of the participating countries.

		ClinRO, Its Questionnaire Version, and a Clinical Amnesia Test						PROMs																PerfO					
		GOSE *		GOSE-Q *		GOAT		GAD-7		PHQ-9		PCL-5 *		RPQ		QOLIBRI *		QOLIBRI-OS *		SF-36v2		SF-12v2 *		RAVLT		TMT A/B		CANTAB (Subtests)	
No.	Language	*T*	*LV*	*T*	*LV*	*T*	*LV*	*T*	*LV*	*T*	*LV*	*T*	*LV*	*T*	*LV*	*T*	*LV*	*T*	*LV*	*T*	*LV*	*T*	*LV*	*T*	*LV*	*T*	*LV*	*T*	*LV*
1	Arabic (for Israel)	✓	✓	✓	✓	✓	✓	-	-	-	-	✓	✓	✓	✓	✓	✓	✓	✓	-	-	-	-	✓	✓	✓	✓	1,3,4,5,6	1,3,4,5,6
2	Bosnian/Croatian/Serbian	✓	✓	✓	✓	✓	✓	✓	✓	-	-	✓	✓	✓	✓	✓	✓	✓	✓	-	-	-	-	✓	✓	✓	✓	1,3,4,5,6	1,3,4,5,6
3	Bulgarian	✓		✓		✓		-	-	-	-	✓		✓		✓		✓		-	-	-	-	✓		✓		1,2,3,4,5,6	
4	Czech	✓		✓		✓		-	-	-	-	✓		✓		✓		✓		-	-	-	-	✓		-	-	1,4,5	
5	Danish	-	-	✓	✓	✓	✓	-	-	-	-	✓	✓	-	-	-	-	-	-	-	-	-	-	✓	✓	-	-	1,2,3,4,5,6	1,2,3,4,5,6
6	Dutch	-	-	✓	✓	✓	✓	-	-	-	-	✓	✓	✓	✓	-	-	-	-	-	-	-	-	✓	✓	✓	✓	1,3	1,3
7	Finnish	-	-	✓	✓	✓	✓	-	-	-	-	✓	✓	✓	✓	-	-	-	-	-	-	-	-	✓	✓	✓	✓	1,2,3,4,5,6	1,2,3,4,5,6
8	French	-	-	✓	✓	✓	✓	-	-	-	-	✓	✓	✓	✓	-	-	-	-	-	-	-	-	✓	✓	✓	✓	1,3	1,3
9	German	-	-	✓	✓	✓	✓	-	-	-	-	✓	✓	-	-	-	-	-	-	-	-	-	-	✓	✓	✓	✓	1	1
10	Hebrew	✓	✓	✓	✓	✓	✓	-	-	-	-	✓	✓	✓	✓	✓	✓	✓	✓	-	-	-	-	✓	✓	✓	✓	1,3,4,5,6	1,3,4,5,6
11	Hungarian	✓	✓	✓	✓	✓	✓	-	-	-	-	✓	✓	✓	✓	✓	✓	✓	✓	-	-	-	-	✓	✓	✓	✓	1,3,4,5,6	1,3,4,5,6
12	Italian	✓	✓	✓	✓	✓	✓	-	-	-	-	✓	✓	✓	✓	-	-	-	-	-	-	-	-	✓	✓	✓	✓	1,3	1,3
13	Latvian	✓	✓	✓	✓	✓	✓	✓	✓	✓	✓	✓	✓	✓	✓	✓	✓	✓	✓	-	-	-	-	✓	✓	✓	✓	1,2,3,4,5,6	1,2,3,4,5,6
14	Lithuanian	✓	✓	✓	✓	✓	✓	-	-	-	-	✓	✓	✓	✓	✓	✓	✓	✓	-	-	-	-	✓	✓	✓	✓	1,2,3,4,5,6	1,2,3,4,5,6
15	Norwegian	-	-	✓	✓	✓	✓	-	-	-	-	-	-	-	-	-	-	-	-	-	-	-	-	✓	✓	✓	✓	1,2,3,4,5,6	1,2,3,4,5,6
16	Romanian	✓	✓	✓	✓	✓	✓	-	-	-	-	✓	✓	✓	✓	✓	✓	✓	✓	-	-	-	-	✓	✓	✓	✓	1,2,4,5	1,2,4,5
17	Russian (for Israel)	✓	✓	✓	✓	✓	✓	-	-	-	-	✓	✓	✓	✓	✓	✓	✓	✓	-	-	-	-	✓	✓	✓	✓	1,4,5	1,4,5
18	Slovakian	✓	✓	✓	✓	✓	✓	-	-	-	-	✓	✓	✓	✓	✓	✓	✓	✓	-	-	-	-	✓	✓	✓	✓	1,4,5	1,4,5
19	Spanish	✓	✓	✓	✓	✓	✓	-	-	-	-	✓	✓	✓	✓	✓	✓	✓	✓	-	-	-	-	✓	✓	✓	✓	1	1
20	Swedish	✓	✓	✓	✓	✓	✓	-	-	-	-	✓	✓	✓	✓	-	-	-	-	-	-	-	-	✓	✓	-	-	1,2,3,4,5,6	1,2,3,4,5,6

Note: ✓ = instruments translated and linguistically validated for the CENTER-TBI study; — = already existing translated and linguistically validated instruments; empty cells or missing CANTAB subtests numbers = no linguistic validation was performed; numbers for CANTAB subtests = already existing translations. * Instruments marked with an asterisk were selected as core instruments (GOSE, QOLIBRI-OS, SF-12v2) [30], complemented by the GOSE-Q, the PCL-5, and the QOLIBRI, and were translated by two translators and cognitively debriefed. ClinROs = clinician-reported outcome instrument; PROMs = patient-reported outcome measures; PerfOs = performance-based outcome instruments; *T* = translation steps performed; see Figure 1 (for Arabic and Russian, all steps except for cognitive debriefing were performed; for Bulgarian, all instruments underwent at least the first harmonization; for Czech, only forward translations were carried out); LV = linguistic validation; GOSE = Glasgow Outcome Scale—Extended; GOSE-Q = Glasgow Outcome Scale—Extended questionnaire version; GOAT = Galveston Orientation Amnesia Test; GAD-7 = Generalized Anxiety Disorder 7 Items Questionnaire; PHQ-9 = Patient Health Questionnaire 9; PCL-5 = Posttraumatic Stress Disorder Checklist; RPQ = Rivermead Post-Concussion Symptoms questionnaire; QOLIBRI = Quality of Life after Brain Injury Scale; QOLIBRI-OS = Quality of Life after Brain Injury—Overall Scale; SF-36v2 = Short Form Health Survey—Version 2; SF-12v2 = 12-Item Short Form Survey—Version 2; RAVLT = Rey Auditory Verbal Learning Test; TMT-A/B = Trail-Making Test A, B; CANTAB = Cambridge Neuropsychological Test Automated Battery; (1) RTI = reaction time, (2) SWM = spatial working memory, (3) PAL = paired associate learning, (4) RVP = rapid visual processing, (5) AST = attention switching task, (6) SOC = stockings of Cambridge.

**Table 4:** Translations, *linguistic* and *full linguistic* validations for the instruments administered in the CENTER-TBI study.

Instrument	Translations		Linguistic Validations		Full Linguistic Validations (Including Cognitive Debriefings)	
	*N*	*%*	*N*	%	*N*	%
**ClinRO, its questionnaire version, and a clinical amnesia test**	**54**	**100%**	**48**	**89%**	**26**	**54%**
GOSE	14	100%	12	86%	10	83%
GOSE-Q	20	100%	18	90%	16	89%
GOAT	20	100%	18	90%	-	-
**PROMs**	**63**	**100%**	**55**	**87%**	**31**	**56%**
GAD-7	2	100%	2	100%	-	-
PHQ-9	1	100%	1	100%	-	-
PCL-5	19	100%	17	89%	15	88%
RPQ	17	100%	15	88%	-	-
QOLIBRI	12	100%	10	83%	8	80%
QOLIBRI-OS	12	100%	10	83%	8	80%
**PerfOs**	**120**	**100%**	**108**	**90%**	**-**	**-**
CANTAB	83	100%	74	89%	-	-
RAVLT	20	100%	18	90%	-	-
TMT-A, B	17	100%	16	94%	-	-
**Total**	**237**	**100%**	**211**	**89%**	**57**	**27%**

Note: *N* = number of translations or linguistic validations, % = percentage, “-” = no translation or linguistic validation performed. Translations = overall number and percentage of performed translations; linguistic validation = overall number of performed linguistic validations and percentage in relation to all established translations; *full linguistic validations* include cognitive debriefings, *linguistic validations* do not; here, the overall number of *full linguistic validations* performed and percentage in relation to all performed *linguistic validations* is reported. Bold are for better readability.

**Table 5:** Average (mean and median) number of differences between the original English version and the first harmonized version.

			Average Number of Changes							
			Mean				Median			
Measure	Text Elements	No.	S	C	I/P	S/G	S	C	I/P	S/G
**ClinRO, Its Questionnaire Version, and a Clinical Amnesia Test**										
GOSE (10 translations)	*In*	11	47	3%	5%	9%	46%	0%	5%	0%
	*I*	19	24%	2%	4%	7%	17%	0%	0%	5%
	*R*	15	17%	1%	5%	2%	13%	0%	0%	0%
GOSE-Q (16 translations)	*In*	2	97%	22%	20%	23%	50%	0%	0%	0%
	*I*	14	18%	8%	7%	6%	14%	7%	4%	3%
	*R*	42	9%	1%	3%	3%	2%	0%	1%	0%
GOAT (16 translations)	*In*	8	13%	2%	8%	10%	6%	0%	0%	0%
	*I*	13	11%	4%	5%	3%	8%	0%	0%	0%
	*R*	-	-	-	-	-	-	-	-	-
PROMs *										
GAD-7 (2 translations)	*In*	1	0%	50%	0%	0%	0%	50%	0%	0%
	*I*	7	0%	0%	0%	0%	0%	0%	0%	0%
	*R*	5	0%	0%	0%	0%	0%	0%	0%	0%
PHQ-9 (1 translation)	*In*	1	0%	100%	0%	0%	0%	100%	0%	0%
	*I*	10	0%	10%	0%	0%	0%	10%	0%	0%
	*R*	5	0%	0%	0%	0%	0%	0%	0%	0%
PCL-5 (15 translations)	*In*	1	60%	20%	0%	20%	0%	0%	0%	0%
	*I*	21	19%	2%	8%	7%	14%	0%	0%	5%
	*R*	5	12%	0%	3%	0%	0%	0%	0%	0%
RPQ (13 translations)	*In*	1	146%	46%	31%	46%	146%	0%	0%	0%
	*I*	17	10%	2%	1%	3%	6%	0%	0%	0%
	*R*	5	14%	0%	2%	3%	20%	0%	0%	0%
QOLIBRI (8 translations)	*In*	7	22%	7%	11%	25%	18%	0%	5%	29%
	*I*	37	10%	1%	4%	6%	5%	0%	5%	7%
	*R*	5	3%	0%	0%	0%	0%	0%	0%	0%
QOLIBRI-OS (8 translations)	*In*	1	50%	50%	25%	75%	0%	0%	0%	88%
	*I*	6	8%	0%	4%	2%	4%	0%	0%	0%
	*R*	5	3%	0%	3%	0%	0%	0%	0%	0%
PerfO *										
RAVLT (16 translations)	*In*	5	71%	13%	11%	33%	60%	0%	0%	0%
	*I*	45	7%	0%	1%	0%	6%	0%	0%	0%
	*R*	-	-	-	-	-	-	-	-	-
TMT A/B (14 translations)	*In*	6	58%	11%	17%	12%	42%	17%	8%	14%
	*I*	38	1%	0%	0%	0%	0%	0%	0%	0%
	*R*	-	-	-	-	-	-	-	-	-

Note: No. = Number of text elements; In = Instructions: average number of modifications between the original English version and the first harmonization (average in %, i.e., number of differences in relation to the total number of the respective text elements divided by the number of translations); I = Items: modifications in items; R = Response categories: modifications (if applicable, otherwise “-“); S/G = Syntactic/Grammatical level; C = Cultural level; I/P = Idiomatic/Pragmatic; ClinRO = clinician-reported outcome instrument; PROMs = patient-reported outcome measures; PerfOs = performance-based outcome instruments; GOSE = Glasgow Outcome Scale—Extended; instructions of the GOSE include introduction (1), commentary on the questions (9), and scoring (1); GOSE-Q = Glasgow Outcome Scale—Extended questionnaire version; instructions include introduction and header (1) and explanatory example for the item 9; different types of responses (dichotomous yes/no and polytomous item-related responses) result in 42 elements; GOAT = Galveston Orientation Amnesia Test (no response categories); GAD-7 = Generalized Anxiety Disorder 7 Items Questionnaire; PHQ-9 = Patient Health Questionnaire 9 Items; PCL-5 = Posttraumatic Stress Disorder Checklist; RPQ = Rivermead Post-Concussion Symptoms questionnaire; QOLIBRI = Quality of Life after Brain Injury Scale; instructions to the two parts (2) and five subscales (5); QOLIBRI-OS = Quality of Life after Brain Injury—Scale; RAVLT = Rey Auditory Verbal Learning Test; here, words are treated as items (5 × 15 = 45) (no response categories); instructions: introduction (1), explanations on the three trials (3), and the summary table for evaluation of the test result (1); TMT-A/B = Trail-Making Test A, B; in the TMT-A/B, letters and numbers are treated as items, there are no response categories; instructions of the TMT-A, B include introduction (1), explanation on the trial A (1) and trial B (1), trial B test (1), scoring (1), and hands check (1); CANTAB = Cambridge Neuropsychological Test Automated Battery. Cognitive debriefings and international harmonization of the Arabic, Russian, Bulgarian, and the Czech translations were not carried out and are therefore not reported. * Excluded from analyses, as translations of the SF-36v2/-12v were obtained from Optum. CANTAB analyses are not presented here, because in the meantime only updated versions from Cambridge Cognition can be used. Therefore, these are not available on the CENTER-TBI website.

**Table 6:** Average comprehension rates per language and outcome instrument in the cognitive debriefings.

		GOSE				GOSE-Q				PCL-5				QOLIBRI				QOLIBRI-OS			
No.	Language	*N*	IN	I	R	*N*	IN	I	R	*N*	IN	I	R	*N*	IN	I	R	*N*	IN	I	R
1	Arabic (for Israel)	-	-	-	-	-	-	-	-	-	-	-	-	-	-	-	-	-	-	-	-
2	Bosnian/Croatian/Serbian	3	100%	100%	100%	6	83%	98%	88%	6	83%	85%	83%	6	67%	92%	100%	6	67%	81%	100%
3	Bulgarian	-	-	-	-	-	-	-	-	-	-	-	-	-	-	-	-	-	-	-	-
4	Czech	-	-	-	-	-	-	-	-	-	-	-	-	-	-	-	-	-	-	-	-
5	Danish	*	*	*	*	6	100%	100%	100%	6	100%	100%	100%	*	*	*	*	*	*	*	*
6	Dutch	*	*	*	*	3	100%	93%	95%	3	100%	95%	100%	*	*	*	*	*	*	*	*
7	Finnish	*	*	*	*	4	100%	98%	100%	6	100%	99%	100%	*	*	*	*	*	*	*	*
8	French	*	*	*	*	3	0%	93%	74%	3	33%	92%	98%	*	*	*	*	*	*	*	*
9	German	*	*	*	*	3	67%	100%	100%	3	100%	80%	100%	*	*	*	*	*	*	*	*
10	Hebrew	2	100%	100%	100%	4	100%	100%	100%	4	100%	100%	100%	4	88%	96%	100%	4	100%	100%	100%
11	Hungarian	2	100%	89%	100%	6	100%	100%	100%	6	100%	96%	100%	6	100%	100%	100%	6	100%	83%	83%
12	Italian	6	100%	91%	100%	6	100%	100%	100%	6	100%	100%	100%	*	*	*	*	*	*	*	*
13	Latvian	10	100%	100%	100%	10	100%	100%	100%	10	100%	100%	100%	10	100%	100%	100%	10	100%	100%	100%
14	Lithuanian	6	100%	100%	100%	6	100%	100%	100%	6	100%	100%	100%	6	100%	100%	100%	6	100%	86%	100%
15	Norwegian	*	*	*	*	6	100%	99%	87%	*	*	*	*	*	*	*	*	*	*	*	*
16	Romanian	3	100%	100%	100%	6	100%	100%	90%	6	100%	100%	100%	6	100%	100%	81%	6	100%	100%	100%
17	Russian (for Israel)	-	-	-	-	-	-	-	-	-	-	-	-	-	-	-	-	-	-	-	-
18	Slovakian	3	100%	86%	97%	6	67%	98%	100%	6	100%	94%	90%	6	92%	98%	100%	6	83%	100%	33%
19	Spanish	3	67%	99%	92%	6	67%	88%	90%	6	100%	81%	100%	10	100%	97%	100%	10	90%	83%	100%
20	Swedish	3	0%	96%	93%	6	83%	70%	76%	6	83%	92%	100%	*	*	*	*	*	*	*	*
	**Average**	**4**	**87%**	**96%**	**98%**	**5**	**85%**	**96%**	**94%**	**6**	**93%**	**94%**	**98%**	**7**	**93%**	**98%**	**98%**	**7**	**93%**	**92%**	**90%**

Note: IN = instructions; I = items, R = item responses; — = no cognitive debriefing performed; * = already existing validated instruments not requiring cognitive debriefing. For the GOSE, mostly clinical personnel were interviewed. Average = the overall average number of participants. Translations are available for the following languages, but no cognitive debriefings: Arabic, Bulgarian, Czech, and Russian. *N* = number of cognitive debriefings performed, and average comprehension rates per instrument. Bold is for better readability.

**Table 7:** Translational issues.

Instrument	Language	Type of Issue	Text Element	Description of the Problems by Target Language Translators	Solution ^1^
GOSE	Italian	Linguistic	Instruction/heading: Date of **injury**	Difficulties with translation of “**injury**” vs. “**trauma**”	Term “**trauma**” selected
GOSE-Q	Finnish	Cultural	Item 7 (response 2): Looking after family	“**Looking after family**” seems to be rather untypical for the Finnish society/culture (exception: maternity or paternity leave, homemaker)	Response extended by “**e.g., maternity or paternity leave**”
	Hebrew	Cultural	Item 9 (examples for social and leisure activities): going out to a pub or club, visiting friends, going to the cinema or **bingo**, going out for a walk, attending a football match, taking part in sport.	Playing **bingo** is rather untypical for Hebrew society/culture	Term “**bingo**” replaced by “**going out to a restaurant**”
	Norwegian	Cultural	Item 9 (examples for social and leisure activities): going out to a **pub** or club, visiting friends, going to the cinema or bingo, going out for a walk, attending a football match, taking part in sport.	Going to **pubs** is rather untypical for Norwegian society/culture	Term “**pub**” replaced by “**café**”
	Swedish	Linguistic	Item 12: As a result of your injury are there now problems in how you get on with friends or relatives? (response 1): **Things** are still much the same	The term “**things**” cannot be used in Swedish in that way	Term “**relationships**” related to the question selected
PCL-5	Danish, Latvian, Croatian/ Bosnian/ Serbian	Linguistic	Item 21: When you responded to the questions in this questionnaire, were your answers in reference to the stressful experience which caused your **traumatic brain injury**?	Difficulties with translation of the term “**injury**” (“**injury**” vs. “**trauma**”, “**head injury**”, or “**accident**”)	The closest meaning to the term “**brain injury**” was selected in each language
	Arabic	Linguistic	Item 17: Being “superalert” or watchful or **on guard**?	Difficulties with translation of “**on guard**”	Corrected translation of “**on guard**” was implemented
RPQ	Finnish	Linguistic/Cultural	Introduction: We would like to know if **you** now suffer any of the symptoms given below. Because many of these symptoms occur normally, we would like **you** to compare **yourself** now with before the accident.	Comment on the translation of the term “**you**” (using pronominal courtesy form would be more appropriate)	**Pronominal courtesy form** was implemented
QOLIBRI	Romanian	Linguistic	Part F, item 5: **Overall**, how bothered are you by the effects of your brain injury?	Difficulties with translation of “**overall**” vs. “**in general**”	Term “**overall**” replaced by “**in general**”
		Linguistic	All items	No female forms of verbs were available	Female and male forms implemented
GOAT	Finnish	Cultural	Item 2a: Where are you now (**city**)?	“Finland is mostly rural, there are a lot of municipalities without towns”	Term “city” replaced by “**place**”

Note: ^1^ The solution was provided after discussion between the patients, the healthy individuals, the core team and the translation teams in the target languages. Bold is for better readability.

**Table 8:** Average (mean and median) number of differences between the first harmonized and the final internationally harmonized translations.

			Average Number of Changes								Examples
			Mean				Median				
Measure	Text Elements	No.	S	C	I/P	S/G	S	C	I/P	S/G	
**ClinRO, its questionnaire version, and a clinical amnesia rating**											
GOSE (10 translations)	*In*	11	31%	2%	10%	20%	18%	0%	9%	9%	**S**: Use of synonyms to find the closest possible, but not a literal, translation, e.g., “injury” vs. “trauma”, “they” vs. “he/she”. **C**: Use of courtesy (West, Middle, East, and Southern European languages) vs. informal (especially Northern European languages) pronominal form and gender-appropriate language (only emerged during harmonization in some translations). **I/P**: Use of appropriate expressions common for the target languages, e.g., “as it appears”. **S/G**: Use of appropriate sentence structures and grammar suitable for the target languages, e.g., word order and spelling.
	*I*	19	22%	2%	1%	15%	21%	0%	0%	13%	
	*R*	15	9%	1%	1%	5%	7%	0%	0%	6%	
GOSE-Q (16 translations)	*In*	2	107%	9%	7%	23%	50%	0%	0%	0%	**S**: Use of synonyms to find the closest possible, but not a literal, translation, e.g., “injury” vs. “trauma”. **C**: Use of courtesy (West, Middle, East, and Southern European languages) vs. informal (especially Northern European languages) pronominal form and gender-appropriate language; use of examples suitable for the target language countries, e.g., “playing bingo” vs. “going out to restaurant” (only emerged during harmonization in some translations). **I/P**: Use of appropriate translations of phrases “at least half as often” and “less than half as often”. **S/G**: Use of appropriate sentence structures, e.g., word order.
	*I*	14	11%	1%	0%	10%	4%	0%	0%	7%	
	*R*	42	7%	6%	1%	3%	2%	0%	0%	2%	
GOAT (16 translations)	*In*	8	10%	6%	3%	10%	0%	0%	0%	0%	**S**: Use of synonyms to find the closest possible, but not a literal, translation, e.g., “injury” vs. “trauma” or “accident”. **C**: Use of courtesy (West, Middle, East, and Southern European languages) vs. informal (especially Northern European languages) pronominal form and gender-appropriate language (only emerged during harmonization in some translations); use of time formats most common to the target language countries, e.g., “am/pm” seem to be uncommon in most languages/countries. **I/P**: Use of appropriate expressions common to the target languages in everyday use, e.g., “Where are you now?”. **S/G**: Use of appropriate sentence structures and grammar suitable for the target languages, e.g., word order and spelling.
	*I*	13	16%	0%	7%	9%	8%	0%	0%	0%	
	*R*	-	-	-	-	-	-	-	-	-	
PROMs *											
GAD-7 (2 translations)	*In*	1	0%	50%	0%	0%	0%	50%	0%	0%	**S**: - **C**: Use of courtesy pronominal form and gender-appropriate language (only emerged during harmonization in some translations). **I/P**: - **S/G**: Use of appropriate sentence structures and grammar suitable for the target languages, e.g., comma placement and spelling.
	*I*	7	0%	0%	0%	0%	0%	0%	0%	0%	
	*R*	5	0%	0%	0%	14%	0%	0%	0%	14%	
PHQ-9 (1 translation)	*In*	1	0%	0%	0%	0%	0%	0%	0%	0%	**S**: - **C**: - **I/P**: - **S/G**: Use of appropriate sentence structures and grammar suitable for the target languages, e.g., comma placement and spelling.
	*I*	10	0%	0%	0%	20%	0%	0%	0%	20%	
	*R*	5	0%	0%	0%	0%	0%	0%	0%	0%	
PCL-5 (15 translations)	*In*	1	100%	7%	7%	47%	0%	0%	0%	0%	**S**: Use of synonyms to find the closest possible, but not a literal, translation, e.g., “stressful” or “disturbing” experience. **C**: Use of courtesy pronominal form and gender-appropriate language (only emerged during harmonization in some translations). **I/P**: Use of appropriate expressions common to the target languages in everyday use, e.g., “Being ‘superalert’ or watchful or on guard”. **S/G**: Use of appropriate tense, sentence structures and grammar suitable for the target languages, e.g., translating of verbs in present continuous (e.g., “being”, “having”, and “feeling”).
	*I*	21	26%	1%	4%	14%	19%	0%	0%	14%	
	*R*	5	11%	0%	1%	7%	0%	0%	0%	0%	
RPQ (13 translations)	*In*	1	123%	23%	23%	77%	0%	0%	0%	77%	**S**: Use of synonyms to find the closest possible, but not a literal, translation of the terms, e.g., “injury” vs. “trauma”, appropriate translation of “poor concentrations” or “forgetfulness”. **C**: Use of courtesy pronominal form (only emerged during harmonization in some translations). **I/P**: Use of appropriate expressions, e.g., in translating response category “no more of a problem (than before)”. **S/G**: Use of appropriate sentence structures, tense, and grammar suitable for the target languages, e.g., translating of verbs in present continuous (e.g., “being” and “feeling”).
	*I*	17	6%	1%	3%	4%	6%	0%	0%	4%	
	*R*	5	9%	0%	3%	6%	0%	0%	0%	0%	
QOLIBRI (8 translations)	*In*	7	14%	2%	0%	7%	14%	0%	0%	0%	**S**: Use of synonyms to find the closest possible, but not a literal, translation of the terms, e.g., “brain injury” vs. “trauma”, “How satisfied are you...” and “How bothered are you...”. **C**: Use of courtesy pronominal form and gender-appropriate language (only emerged during harmonization in some translations). **I/P**: Use of appropriate expressions, e.g., to be “in charge of your own life”. **S/G**: Use of appropriate sentence structures and grammar suitable for the target languages, e.g., comma placement and spelling.
	*I*	37	10%	0%	1%	5%	9%	0%	0%	5%	
	*R*	5	20%	0%	0%	13%	10%	0%	0%	0%	
QOLIBRI-OS (8 translations)	*In*	1	25%	13%	38%	38%	0%	0%	0%	0%	**S**: Use of synonyms to find the closest possible, but not a literal, translation of the terms, e.g., “brain injury” vs. “trauma”, “future prospects” vs. “plans for the future”. **C**: Use of courtesy pronominal form and gender-appropriate language (only emerged during harmonization in some translations). **I/P**: Use of appropriate expressions common in everyday use, e.g., “don’t hesitate to ask for help”. **S/G**: Use of appropriate sentence structures and grammar suitable for the target languages, e.g., comma placement and spelling.
	*I*	6	13%	4%	2%	4%	6%	0%	0%	0%	
	*R*	5	5%	0%	3%	3%	0%	0%	0%	0%	
PerfO *											
RAVLT (16 translations)	*In*	5	81%	8%	9%	41%	30%	0%	0%	40%	**S**: Use of synonyms to find the closest possible, but not a literal, translation of the instructions. **C**: Use of courtesy pronominal form and gender-appropriate language (only emerged during harmonization in some translations); use of language- and/or culture-specific terms (e.g., “church” vs. “synagogue”). **I/P**: Selection of suitable words more frequently used in everyday life, e.g., “tool” or “cake”. **S/G**: Use of appropriate sentence structures and grammar suitable for the target languages, e.g., comma placement and spelling especially in the instructions.
	*I*	45	3%	0%	2%	1%	3%	0%	0%	0%	
	*R*	*-*	-	-	-	-	-	-	-	-	
TMT A/B (14 translations)	*In*	6	14%	2%	5%	19%	0%	0%	0%	0%	**S**: Use of synonyms to find the closest possible, but not a literal, translation of the instructions, e.g., “participant” or “examiner”. **C**: Use of courtesy pronominal form and gender-appropriate language (only emerged during harmonization in some translations). **I/P**: Selection of suitable words more frequently used in everyday life, e.g., to be “sure about” something. **S/G**: Use of appropriate sentence structures and grammar suitable for the target languages, e.g., comma placement and spelling, especially in the instructions.
	*I*	38	0%	0%	0%	0%	0%	0%	0%	0%	
	*R*	-	-	-	-	-	-	-	-	-	

Note: No. = No. of text elements; In = average modifications between the first harmonization and the final version in instructions (average in %, i.e., number of differences relative to the total number of the respective text elements divided by the number of translations); I = modifications in items; R = modifications in response categories (if applicable, otherwise “-“); S = syntactic level; C = cultural level; I/P = idiomatic/pragmatic level; S/G = syntactical/grammatical level; ClinRO = clinician-reported outcome instrument; PROM = patient-reported outcome measures; PerfO = performance-based outcome instruments; GOSE = Glasgow Outcome Scale—Extended; instructions of the GOSE include introduction (1), commentary on the questions (9), and scoring (1); GOSE-Q = Glasgow Outcome Scale—Extended questionnaire version; instructions of the GOSE-Q include introduction and header (1) and explanatory example for the item 9; different types of responses (dichotomous yes/no, including extensions for GOSE scoring) and polytomous item-related responses result in 42 elements; GOAT = Galveston Orientation Amnesia Test; the GOAT has no response categories; GAD-7 = Generalized Anxiety Disorder 7 Items Questionnaire; PHQ-9 = Patient Health Questionnaire 9; PCL-5 = Posttraumatic Stress Disorder Checklist; RPQ = Rivermead Post-Concussion Symptoms questionnaire; QOLIBRI = Quality of Life after Brain Injury Scale; instructions of the QOLIBRI include introduction to the two parts (2) and five subsections (5); QOLIBRI-OS = Quality of Life after Brain Injury—Overall Scale; RAVLT = Rey Auditory Verbal Learning Test; in the RAVLT, words are treated as items (5 × 15 = 45), there are no response categories; instructions of the RAVLT include introduction (1), explanations on the three trials (3), and the summary table for evaluation of the test result (1); TMT-A/B = Trail-Making Test A, B; in the TMT-A/B, letters and numbers are treated as items, there are no response categories; instructions of the TMT-A, B include introduction (1), explanation on the trial A (1) and trial B (1), trial B test (1), scoring (1), hands check (1); CANTAB = Cambridge Neuropsychological Test Automated Battery. * Excluded from analysis as translations of the SF-36v2/-12v were obtained from Optum. CANTAB analyses are not presented here as in the meantime only updated versions from Cambridge Cognition can be used. Therefore, these are not available on the CENTER-TBI website. Cognitive debriefings and international harmonization of the Arabic, Russian, Bulgarian, and the Czech translations were not carried out and are thus not reported.

## Data Availability

All relevant data are available upon request from CENTER-TBI, and the authors are not legally allowed to share it publicly. The authors confirm that they received no special access privileges to the data. CENTER-TBI is committed to data sharing and in particular to responsible further use of the data. Hereto, we have a data-sharing statement in place: https://www.center-tbi.eu/data/sharing, accessed on 16 April 2021. The CENTER-TBI Management Committee, in collaboration with the General Assembly, established the Data Sharing policy, and Publication and Authorship Guidelines to assure correct and appropriate use of the data as the dataset is hugely complex and requires help of experts from the Data Curation Team or Bio- Statistical Team for correct use. This means that we encourage researchers to contact the CENTER-TBI team for any research plans and the Data Curation Team for any help in appropriate use of the data, including sharing of scripts. Requests for data access can be submitted online: https://www.center-tbi.eu/data, accessed on 16 April 2021. The complete Manual for data access is also available online: https://www.center-tbi.eu/files/SOP-Manual-DAPR-20181101.pdf, accessed on 16 April 2021.

## Supplementary Materials

The following are available online at https://www.mdpi.com/article/10.3390/jcm10132863/s1, Table S1: Comparisons between the original English versions and the first draft translations of one ClinRO, its questionnaire version, a clinical amnesia test, PROMs, and PerfO instruments, Table S2: Comparisons between the first draft translations and the final target language versions of one ClinRO, its questionnaire version, a clinical amnesia test, PROMs, and PerfO instruments.

## Appendix A. Linguistic Validation Group

**Name**	**Affiliation**
Abu Shkara Ramiz	Department of Neurosurgery, Hadassah-Hebrew University Medical Center, Jerusalem, Israel
Alshafai Nabeel	Neurosurgical Academy, Toronto, Canada
Andelic Nada	Department of Physical Medicine and Rehabilitation, Oslo University Hospital, Oslo, Norway and Faculty of Medicine, Institute of Health and Society, Research Centre for Habilitation and Rehabilitation Models and Services (CHARM), University of Oslo, Oslo, Norway
Azouvi Philippe	Physical and Rehabilitation Medicine, Hospital Raymond Poincaré, Garches, France
Bachvarova Maya	Institute for Medical Psychology and Medical Sociology, University Medical Center Göttingen, Göttingen, Germany
Bakx Wilbert	Department of brain injury, Adelante, Adult rehabilitation, Hoensbroek, The Netherlands
Bar Sapir Peleg	no affiliation
Björkdahl Ann	Ersta Sköndal Bräcke University College, Institute of Social Science, campus Bräcke, Gothenburg, Sweden
Branca Enrica	Institute for Medical Psychology and Medical Sociology, University Medical Center Göttingen, Göttingen, Germany
Brazinova Alexandra	Faculty of Medicine, Comenius University, Bratislava, Slovak Republic
Chatelle Camille	Louvain Bionics, Université catholique de Louvain, Ottignies-Louvain-la-Neuve, Belgium
Dahyot-Fizelier Claire	Department of Anesthesia and Intensive Care, University Hospital of Poitiers, INSERM U1070, University of Poitiers, Poitiers, France
Del Bianco Silvia	Neurointensive Care Unit, San Gerardo Hospital Monza, Monza, Italy
Furmanov Alex	Neurosurgery ICU, Hadassah Hebrew University Medical Center, Jerusalem, Israel
Godbolt Alison	University Department of Rehabilitation Medicine Stockholm, Danderyd Hospital, Sweden
Gürlich Robert	General University Hospital Kralovske Vinohrady, Prague, Czech Republic
Hedenäs Anna	University Department of Rehabilitation Medicine, Danderyd Hospital, Acquired Brain Injury Unit, Daycare, Stockholm, Sweden
Hoang Stéphane	Institute for Medical Psychology and Medical Sociology, University Medical Center Göttingen, Göttingen, Germany
Kanuscak Martin	Institute for Medical Psychology and Medical Sociology, University Medical Center Göttingen, Göttingen, Germany
Karan Mladen	Department of Neurosurgery, Haukeland University Hospital, Bergen, Norway
Kondziella Daniel	Department of Neurology, Rigshospitalet, Copenhagen University Hospital, Copenhagen, Denmark
Koskinen Sanna	Department of Psychology and Logopedics, University of Helsinki, Helsinki, Finnland
Laleva Maria	Department of Neurosurgery, University Hospital Pirogov, Sofia, Bulgaria
Levi Leon	Rambam Healthcare Campus, Haifa, Israel
Liebertau Pia	Klinik IV, Klinikum Osnabrück, Germany, and Institut für Ethik und Geschichte der Medizin, University Medical Center Göttingen, Göttingen, Germany
Lundgaard Soberg Helene	Department of Physical Medicine and Rehabilitation, Oslo University Hospital, Oslo, Norway
Martino Costanza	Dipartimento Chirurgico e Grandi Traumi, Ospedale M. Bufalini, Cesena, Italy
Menovsky Tomas	Department of Neurosurgery, Antwerp University Hospital, Antwerp, Belgium
Milinkovic Sladana	Clinical Center of Vojvodina, Novi Sad, Serbia
Mondello Stefania	Department of Biomedical and Dental Sciences and Morphofunctional Imaging, University of Messina, Italy
Nelson David	Department of Physiology and Pharmacology, Karolinska Universitetssjukhus, Solna, Sweden
Oistensen Holthe Oyvor	Department of Physical Medicine and Rehabilitation, Oslo University Hospital, Oslo, Norway
Pestovskaya Natalia	Burdenko National Medical Research Center of Neurosurgery (NN Burdenko NMRCN), Moscow, Russia
Pfeiffer Anna	Institute for Medical Psychology and Medical Sociology, University Medical Center Göttingen, Göttingen, Germany
Popescu Codruta	Department of Practical Abilities, Human Science, University of Medicine and Pharmacy Iuliu Hatieganu, Cluj-Napoca, Romania
Potapov Aleksandr	Burdenko National Medical Research Center of Neurosurgery (NN Burdenko NMRCN), Moscow, Russia
Poulsen Ingrid	Department of Neurorehabilitation, TBI Unit, Rigshospitalet, Denmark and Research Unit Nursing and Health Care, Health, Aarhus University, Denmark
Radoi Andreea	Clinical Research Office at BarcelonaBeta Brain Research Center, Barcelona, Spain
Ragauskas Arminas	Health Telematics Science Institute, Kaunas University of Technology, Kaunas, Lithuania
Ramin Irina	Institute for Medical Psychology and Medical Sociology, University Medical Center Göttingen, Göttingen, Germany
Ramin Petr	Institute for Medical Psychology and Medical Sociology, University Medical Center Göttingen, Göttingen, Germany
Ramin Stanislav	Institute for Medical Psychology and Medical Sociology, University Medical Center Göttingen, Göttingen, Germany
Reggi Valeria	University of Brescia, Brescia, Italy and University of Bologna, Bologna, Italy
Reith Florence	Department of Neurosurgery, Sir Charles Gairdner Hospital, Perth, WA, Australia
Ribbers Gerard	Department of Rehabilitation Medicine, Erasmus MC, University Medical Center Rotterdam, Rotterdam, the Netherlands
Rocka Saulius	Clinic of Neurology and Neurosurgery, Vilnius University, Vilnius, Lithuania
Rosenlund Sorensen Christina	Department of Neurosurgery, Odense University Hospital, Odense, Denmark
Rosenthal Guy	Department of Neurosurgery, Hadassah-Hebrew University Medical Center, Jerusalem, Israel
Sahuquillo Juan	Department of Neurosurgery, Vall d’Hebron University Hospital, Barcelona, Spain
Schäfer Kaja J.	Department of Pediatrics, Klinikum Bremerhaven-Reinkenheide gGmbH, Bremerhaven, Germany
Schou Rico	Neuroanesthesiology and -Intensive Care, University Hospital of Odense, Odense, Denmark
Siponkoski Sini-Tuulu	Cognitive Brain Research Unit, Department of Psychology and Logopedics, University of Helsinki, Helsinki, Finnland
Skandsen Toril	Institute of Neuromedicine and Movement sciences Norwegian University of Science and Technology Specialist in Physical and Rehabilitation Medicine St. Olavs Hospital, Trondheim University Hospital, Norway
Skoett Skare BruunMarie	Danish Dementia Research Centre, Department of Neurology, Rigshospitalet, University of Copenhagen, Denmark
Skowroneck Lukas	Department of Languages, Literature and Communication, University of Utrecht, Utrecht, the Netherlands
Sokkanen Sanna E.	South Karelia Social and Health Care District, University of Jyväskylä, Jyväskylä, Finnland
Sonne E. Morten	Centre of Head and Orthopaedics, Department of Anaesthesiology, Copenhagen University Hospital, Rigshospitalet, Copenhagen, Denmark
Steinbusch Catherine	Adelante Rehabilitation Centre Valkenburg, Valkenburg, the Netherlands
Stocchetti Nino	Dipartimento Fisiopatologia e Trapianti, Fondazione IRCCS Cà Granda Ospedale Maggiore Poloclinico, Milan, Italy
Tamir Idit	Neurosurgery Department, Rabin Medical Center Petach Tikva, Petach Tikva, Israel
Tenovuo Olli	Turku Brain Injury Centre, University of Turku and Turku University Hospital, Turku, Finnland
Teunissen Charlotte	Vrije Universiteit Amsterdam, Amsterdam, The Netherlands
Theodorou Konstantina	Institute for Medical Psychology and Medical Sociology, University Medical Center Göttingen, Göttingen, Germany
Turcsányi Ditta	English and Hungarian as a Foreign Language Teacher
Valeinis Egils	Neurosurgery Clinic, Pauls Stradins Clinical University Hospital, Riga, Latvia
Van der MerschNils	Université Libre de Bruxelles, Bruxelles, Belgium
van Laake-Geelen Charlotte	Adelante Centre of Expertise in Rehabilitation and Audiology, Hoensbroek, The Netherlands
Van Praag Dominique	Department of Psychology, Antwerp University Hospital, Edegem and University of Antwerp, Antwerp, Belgium
Vargiolu Alessia	School of Medicine and Surgery, University of Milano—Bicocca, Milan, Italy
Velinov Nikolay	Department of Neurosurgery for Adults, Clinics of Neurosurgery, University Hospital Pirogov, Novi Sad, Serbia
Vilcinis Rimantas	Department of Neurosurgery, Hospital of Lithuanian University of Health Sciences, Kaunas, Lithuania
Vulekovic Petar	Clinic of Neurosurgery, Clinical Center of Vojvodina, Novi Sad, Serbia

## Appendix B. Instruments Administered in the CENTER-TBI Study

### Appendix B.1. Clinician-Reported Outcome Assessments (ClinRO), Its Clinical Version and a Clinical Amnesia Test

*Glasgow Outcome Scale Extended (GOSE-Interview)* [37]. This clinical rating evaluates patients’ functional status and level of disability. The GOSE consists of 19 items with both dichotomous and polytomous responses measuring different domains (consciousness, independence at home and outside home, work, social and leisure activities, family and friendships, return to normal life, and epilepsy). Functional outcome is rated by a clinician on an eight-point scale (1 = dead, 2 = vegetative state, 3/4 = lower/upper severe disability, 5/6 = lower/upper moderate disability, and 7/8 = lower/upper good recovery).

*Glasgow Outcome Scale Extended—Questionnaire version (GOSE-Q)* [38]. This questionnaire addresses similar aspects to the GOSE interview in a format that a patient can answer with or without help, or a caretaker can complete. The GOSE-Q consists of 14 items with different response formats (from “yes”/“no” to specifically item-related rating scales and response categories). In the CENTER-TBI study, the GOSE-Q was used for data collection via mail or during a visit. The allocation to the respective functional status categories according to the GOSE definition was performed centrally.

*Galveston Orientation Amnesia Test (GOAT)* [39]. This standardized evaluation based on patient responses assesses whether an individual suffers from post-traumatic amnesia (PTA). It comprises ten items measuring orientation, memory for the first event that the participant can recall after the injury (anterograde amnesia), and memory for the last event that the participant can recall from before the injury (retrograde amnesia). The instrument is administered by clinical personnel.

### Appendix B.2. Patient-Reported Outcome Measures (PROMs)

*Generalized Anxiety Disorder 7 (GAD-7)* [40]. With seven items, this self-report tool assesses symptoms of generalized anxiety disorder with a recall period of the past two weeks. The GAD-7 applies a four-point Likert scale (from 0 “not at all” to 3 “nearly every day”).

*Patient Health Questionnaire 9 (PHQ-9)* [41]. This short self-report instrument captures presence and severity of major depression using nine items based on the Diagnostic and Statistical Manual of Mental Disorders, 4th edition (DSM-IV) [59], criteria with a recall period of the past two weeks.

*Posttraumatic Stress Disorder Checklist* (PCL-5) [42]. This self-report scale evaluates 20 posttraumatic stress disorder (PTSD) symptoms with a recall period of one week at the two-week assessment and a recall period of a month for later assessments. The rating is based on the Diagnostic and Statistical Manual of Mental Disorders, 5th edition (DSM-5) [60] and applies a five-point Likert scale (from 0 “not at all” to 4 “extremely”).

*Rivermead Post-Concussion Symptoms Questionnaire (RPQ)* [43]. This self-report questionnaire measures 16 post-concussion symptoms after TBI applying a five-point Likert scale (from 0 “not experienced at all” to 4 = “a severe problem”). Individuals rate how much they suffered from the following symptoms over the last 24 h compared with their condition before the injury: headaches, dizziness, nausea and/or vomiting, noise sensitivity, sleep disturbance, fatigue, irritability, depression, frustration, forgetfulness and poor memory, poor concentration, slow thinking, blurred vision, light sensitivity, double vision, and restlessness. Two additional open questions assess further difficulties experienced after TBI which should be rated on the same scale as other symptoms.

*Quality of Life after Brain Injury Scale (QOLIBRI)* [44,45]. This is a disease-specific instrument for individuals after TBI, assessing HRQOL using 37 items on a five-point Likert scale (from 0 “not at all” to 4 “very”). Based on these items, the following six domains are evaluated: cognition, self, daily life and autonomy, social relationships, emotions, and physical problems.

*Quality of Life after Brain Injury—Overall Scale (QOLIBRI-OS)* [46]. The QOLIBRI-OS is a short screener of disease-specific HRQOL after TBI. It comprises six items, which measure physical conditions, cognition, emotions, daily life and autonomy, social relationships, and current and future prospects, respectively. The QOLIBRI-OS applies a five-point Likert scale (from 0 “not at all” to 4 “very”).

Additionally, two generic PROMs assessing the subjective health status—the 36-item Short Form Health Survey—Version 2 (SF-36v2) [47] and the 12-item Short Form Survey— Version 2 (SF-12v2) [48]—were administered in the CENTER-TBI study. Both measures had already been translated into the target languages and had to be purchased from Optum [52] for one-time use.

### Appendix B.3. Performance-Based Outcome Instruments (PerfO)

*Cambridge Neuropsychological Test Automated Battery (CANTAB)* [49]. The CANTAB is a computer-based, mainly language-independent test battery well suited for administration in multinational settings. The license to use this battery is made available by Cambridge Cognition [61]. Although the CANTAB is language-independent and is administered by trained personnel, participants should receive standardized information on the test procedure. The instructions are provided for each of six subtests and contain explanations and examples for each task (e.g., instructions for practice trials, hints for keyboard usage, prompts, etc.). To ensure the validity of the GOAT translations, this information should be equivalent across the languages. Therefore, the test instructions of the following six CANTAB tests, selected for the application in the CENTER-TBI study, needed to be translated into most languages:

1. Reaction Time (RTI)—The RTI is designed to measure motor and mental response speed, movement time, reaction time, response accuracy, and impulsivity.
2. Spatial Working Memory (SWM)—This test measures the subject’s ability to remember spatial information and requires retention and manipulation of visual-spatial information, working memory, and executive functions.
3. Paired Associate Learning (PAL)—This test assesses conditional learning and episodic visual memory and is primarily sensitive to processes associated with the medial temporal lobe.
4. Rapid Visual Processing (RVP)—The RVP captures visual sustained attention, being sensitive to dysfunctions in the parietal and frontal lobe brain areas.
5. Attention Switching Task (AST)—As a measure of cued attentional set-shifting this test captures executive functioning and cognitive flexibility.
6. Stockings of Cambridge (SOC)—Spatial planning and spatial working memory are assessed with this measure of frontal-lobe functioning.

*Rey Auditory Verbal Learning Test (RAVLT)* [50]. This memory word list evaluates a range of memory functions, including verbal memory, shorter and longer retention of information and learning, and is administered by trained examiners. The instrument consists of two lists (A and B form) comprising 15 words, respectively. In total, there were four versions of the RAVLT, each with two different word lists. Three versions of two RAVLT word lists were administered in the CENTER-TBI study (lists 1 to 3 in 2-week, 3-month, and 6-month outcome assessments, respectively, and lists 3 to 1 at 6-, 12-, and 24-month outcome assessments, respectively).

*Trail-Making Test A, B (TMT-A, B)* [51]. This test measures executive functions, such as visual attention, speed, and mental flexibility, and consists of two parts (A+B). The TMT-A consists of 25 circles filled with numbers (1 to 25), which are to be connected in ascending order (from 1 to 2, from 2 to 3, etc.). The TMT-B includes both numbers (1 to 13) and letters (A to L), which are to be connected in ascending order from a letter to a number (i.e., from 1 to A, from A to 2, from 2 to B, etc.). In both tests, the time is measured, and the result is reported in seconds.
