# Supplementary material for: Translation and Linguistic Validation of Outcome Instruments for Traumatic Brain Injury Research and Clinical Practice: A Step-by-Step Approach within the Observational CENTER-TBI Study
Source: J Clin Med. 2021 Jun 28;10(13):2863. doi: 10.3390/jcm10132863 (PMC8269004; doi:10.3390/jcm10132863)
Supplement: Supplementary file 1 [file jcm-10-02863-s001.zip › jcm-1276004-supplementary.pdf]

Comparisons between the original English versions and the first draft translations of one ClinRO, its questionnaire version, a clinical amnesia test, PROMs, and PerfO instruments

		ClinRO, its questionnaire version, and a clinical rating									PROM																		PerfO					
		GOSE			GOSE-Q			GOAT			GAD-7			PHQ-9			PCL-5			RPQ			QOLIBRI			QOLIBRI-OS			RAVLT			TMT A/B		
		19 items			14 items			10 items			7 items			9+1 items			20+1 items			16 + 2 items			37 items			6 items			Three versions of two word lists, 15 words each			numbers and letters		
Language	Type of differences (*column*) and no. of elements (*row*)	*In*	*I*	*R*	*In*	*I*	*R*	*In*	*I*	*R*	*In*	*I*	*R*	*In*	*I*	*R*	*In*	*I*	*R*	*In*	*I*	*R*	*In*	*I*	*R*	*In*	*I*	*R*	*In*	*I*	*R*	*In*	*I*	*R*
		11	19	15	2	14	42	8	13	0	1	7	5	1	10	5	1	21	5	1	17	5	7	37	5	1	6	5	5	45	0	6	38	0
Bosnian/ Croatian/ Serbian	Semantic	0%	42%	0%	150%	14%	2%	0%	46%		0%	0%	0%	-	-	-	100%	19%	20%	0%	6%	40%	86%	43%	0%	0%	33%	0%	60%	0%		50%	0%	
	Cultural	0%	0%	0%	0%	21%	0%	0%	0%		0%	0%	0%	-	-	-	0%	0%	0%	100%	6%	0%	0%	0%	0%	0%	0%	0%	0%	0%		0%	0%	
	Idiomatic	0%	11%	0%	0%	0%	0%	13%	0%		0%	0%	0%	-	-	-	0%	0%	0%	0%	0%	0%	0%	0%	0%	0%	0%	0%	0%	4%		0%	0%	
	Syntactic/grammatical	0%	16%	0%	50%	14%	0%	0%	15%		0%	0%	0%	-	-	-	0%	29%	0%	0%	12%	0%	29%	8%	0%	100%	0%	0%	0%	0%		17%	0%	
Danish	Semantic	-	-	-	0%	0%	0%	0%	8%		-	-	-	-	-	-	0%	10%	20%	-	-	-	-	-	-	-	-	-	0%	4%		-	-	
	Cultural	-	-	-	0%	0%	0%	0%	0%		-	-	-	-	-	-	0%	0%	0%	-	-	-	-	-	-	-	-	-	0%	0%		-	-	
	Idiomatic	-	-	-	0%	7%	2%	0%	0%		-	-	-	-	-	-	0%	0%	0%	-	-	-	-	-	-	-	-	-	0%	0%		-	-	
	Syntactic/grammatical	-	-	-	0%	0%	0%	0%	0%		-	-	-	-	-	-	0%	5%	0%	-	-	-	-	-	-	-	-	-	0%	0%		-	-	
Dutch	Semantic	-	-	-	100%	29%	14%	0%	0%		-	-	-	-	-	-	0%	14%	60%	100%	6%	20%	-	-	-	-	-	-	20%	4%		0%	8%	
	Cultural	-	-	-	0%	14%	0%	0%	0%		-	-	-	-	-	-	100%	0%	0%	0%	0%	0%	-	-	-	-	-	-	0%	0%		0%	0%	
	Idiomatic	-	-	-	50%	14%	5%	0%	15%		-	-	-	-	-	-	0%	19%	0%	0%	6%	0%	-	-	-	-	-	-	0%	0%		17%	0%	
	Syntactic/grammatical	-	-	-	0%	0%	0%	0%	0%		-	-	-	-	-	-	200%	0%	0%	0%	0%	0%	-	-	-	-	-	-	0%	0%		0%	0%	
Finnish	Semantic	-	-	-	100%	29%	0%	0%	8%		-	-	-	-	-	-	0%	0%	0%	200%	6%	20%	-	-	-	-	-	-	120%	11%		17%	0%	
	Cultural	-	-	-	50%	7%	2%	0%	0%		-	-	-	-	-	-	0%	0%	0%	100%	0%	0%	-	-	-	-	-	-	0%	0%		0%	0%	
	Idiomatic	-	-	-	0%	0%	0%	0%	0%		-	-	-	-	-	-	0%	0%	0%	0%	0%	0%	-	-	-	-	-	-	0%	0%		0%	0%	
	Syntactic/grammatical	-	-	-	50%	7%	0%	0%	0%		-	-	-	-	-	-	0%	5%	0%	0%	0%	0%	-	-	-	-	-	-	80%	0%		33%	0%	
French	Semantic	-	-	-	50%	14%	0%	25%	15%		-	-	-	-	-	-	0%	14%	0%	200%	6%	0%	-	-	-	-	-	-	80%	4%		67%	0%	
	Cultural	-	-	-	50%	14%	0%	0%	8%		-	-	-	-	-	-	0%	5%	0%	0%	6%	0%	-	-	-	-	-	-	20%	0%		17%	0%	
	Idiomatic	-	-	-	0%	7%	5%	0%	23%		-	-	-	-	-	-	0%	14%	0%	100%	6%	20%	-	-	-	-	-	-	0%	2%		0%	0%	
	Syntactic/grammatical	-	-	-	0%	7%	2%	38%	8%		-	-	-	-	-	-	100%	0%	0%	0%	12%	0%	-	-	-	-	-	-	40%	0%		0%	0%	
German	Semantic	-	-	-	200%	79%	62%	113%	38%		-	-	-	-	-	-	300%	52%	0%	-	-	-	-	-	-	-	-	-	380%	16%		283%	0%	
	Cultural	-	-	-	50%	7%	7%	0%	8%		-	-	-	-	-	-	0%	0%	0%	-	-	-	-	-	-	-	-	-	40%	0%		17%	0%	
	Idiomatic	-	-	-	50%	21%	10%	50%	8%		-	-	-	-	-	-	0%	24%	40%	-	-	-	-	-	-	-	-	-	20%	0%		33%	0%	
	Syntactic/grammatical	-	-	-	50%	21%	10%	0%	8%		-	-	-	-	-	-	0%	10%	0%	-	-	-	-	-	-	-	-	-	0%	2%		33%	0%	
Hebrew	Semantic	9%	0%	7%	0%	0%	0%	0%	0%		-	-	-	-	-	-	0%	14%	0%	0%	6%	0%	0%	5%	0%	0%	0%	0%	0%	7%		0%	0%	
	Cultural	0%	0%	0%	0%	0%	0%	0%	0%		-	-	-	-	-	-	0%	0%	0%	0%	0%	0%	0%	0%	0%	0%	0%	0%	0%	0%		0%	0%	
	Idiomatic	0%	0%	0%	0%	0%	0%	0%	0%		-	-	-	-	-	-	0%	0%	0%	0%	0%	0%	0%	0%	0%	0%	0%	0%	0%	0%		0%	0%	
	Syntactic/grammatical	0%	0%	0%	0%	0%	2%	0%	0%		-	-	-	-	-	-	0%	0%	0%	0%	0%	0%	0%	3%	0%	0%	0%	0%	0%	0%		0%	0%	
Hungarian	Semantic	82%	63%	20%	400%	21%	5%	13%	8%		-	-	-	-	-	-	200%	43%	20%	200%	12%	20%	29%	14%	0%	0%	0%	0%	140%	18%		50%	0%	
	Cultural	0%	5%	0%	100%	7%	0%	0%	23%		-	-	-	-	-	-	0%	5%	0%	200%	6%	0%	29%	5%	0%	200%	0%	0%	40%	0%		17%	0%	
	Idiomatic	0%	0%	0%	50%	21%	2%	0%	0%		-	-	-	-	-	-	0%	10%	0%	100%	0%	0%	0%	5%	0%	0%	0%	0%	0%	4%		17%	0%	
	Syntactic/grammatical	0%	0%	0%	0%	0%	0%	13%	0%		-	-	-	-	-	-	0%	0%	0%	0%	0%	0%	0%	0%	0%	0%	0%	0%	0%	0%		17%	3%	
Italian	Semantic	73%	11%	0%	0%	0%	2%	13%	0%		-	-	-	-	-	-	0%	0%	0%	0%	0%	0%	-	-	-	-	-	-	0%	0%		0%	0%	
	Cultural	9%	5%	13%	0%	7%	2%	13%	8%		-	-	-	-	-	-	100%	5%	0%	0%	6%	0%	-	-	-	-	-	-	40%	0%		17%	0%	
	Idiomatic	18%	0%	0%	0%	0%	0%	0%	0%		-	-	-	-	-	-	0%	0%	0%	0%	0%	0%	-	-	-	-	-	-	0%	0%		17%	0%	
	Syntactic/grammatical	0%	0%	7%	0%	0%	0%	0%	0%		-	-	-	-	-	-	0%	5%	0%	0%	0%	0%	-	-	-	-	-	-	0%	0%		0%	0%	
Latvian	Semantic	18%	11%	7%	50%	7%	2%	13%	8%		0%	0%	0%	0%	0%	0%	0%	5%	0%	100%	0%	0%	14%	3%	0%	100%	0%	0%	0%	0%		0%	0%	
	Cultural	0%	0%	0%	50%	7%	0%	13%	8%		100%	0%	0%	100%	10%	0%	100%	5%	0%	100%	0%	0%	14%	3%	0%	100%	0%	0%	20%	0%		17%	0%	
	Idiomatic	0%	0%	0%	0%	0%	0%	0%	0%		0%	0%	0%	0%	0%	0%	0%	0%	0%	0%	0%	0%	0%	0%	0%	0%	0%	0%	0%	0%		0%	0%	
	Syntactic/grammatical	0%	0%	0%	0%	0%	0%	0%	0%		0%	0%	0%	0%	0%	0%	0%	0%	0%	100%	0%	0%	57%	19%	0%	100%	0%	0%	0%	0%		0%	0%	
Lithuanian	Semantic	9%	11%	13%	50%	29%	10%	0%	0%		-	-	-	-	-	-	0%	5%	40%	300%	6%	20%	29%	0%	0%	0%	17%	0%	0%	0%		50%	0%	
	Cultural	0%	0%	0%	0%	0%	0%	0%	0%		-	-	-	-	-	-	0%	0%	0%	0%	0%	0%	0%	0%	0%	0%	0%	0%	0%	0%		17%	0%	
	Idiomatic	0%	0%	0%	0%	0%	0%	0%	0%		-	-	-	-	-	-	0%	0%	0%	0%	0%	0%	14%	5%	0%	0%	0%	0%	0%	0%		0%	0%	
	Syntactic/grammatical	9%	5%	0%	150%	7%	0%	0%	0%		-	-	-	-	-	-	0%	14%	0%	100%	0%	40%	43%	14%	0%	100%	17%	0%	40%	0%		17%	0%	
Norwegian	Semantic	-	-	-	0%	0%	0%	0%	0%		-	-	-	-	-	-	-	-	-	-	-	-	-	-	-	-	-	-	0%	0%		0%	0%	
	Cultural	-	-	-	0%	7%	0%	0%	0%		-	-	-	-	-	-	-	-	-	-	-	-	-	-	-	-	-	-	0%	0%		0%	0%	
	Idiomatic	-	-	-	0%	0%	0%	0%	0%		-	-	-	-	-	-	-	-	-	-	-	-	-	-	-	-	-	-	0%	0%		0%	0%	
	Syntactic/grammatical	-	-	-	0%	0%	0%	0%	0%		-	-	-	-	-	-	-	-	-	-	-	-	-	-	-	-	-	-	40%	0%		0%	0%	
Romanian	Semantic	9%	16%	0%	50%	7%	5%	0%	8%		-	-	-	-	-	-	0%	5%	0%	100%	6%	0%	14%	5%	0%	100%	0%	0%	0%	2%		0%	0%	
	Cultural	9%	0%	0%	50%	14%	5%	0%	8%		-	-	-	-	-	-	0%	5%	0%	0%	6%	0%	14%	3%	0%	100%	0%	0%	20%	0%		17%	0%	
	Idiomatic	0%	0%	0%	50%	14%	0%	0%	8%		-	-	-	-	-	-	0%	0%	0%	0%	0%	0%	0%	3%	0%	0%	0%	0%	0%	0%		0%	0%	
	Syntactic/grammatical	0%	0%	0%	0%	0%	0%	13%	0%		-	-	-	-	-	-	0%	0%	0%	0%	0%	0%	0%	0%	0%	100%	0%	0%	0%	0%		0%	0%	
Slovakian	Semantic	45%	11%	13%	50%	0%	2%	0%	15%		-	-	-	-	-	-	0%	19%	0%	300%	29%	0%	1%	5%	20%	0%	0%	20%	60%	11%		33%	0%	
	Cultural	9%	5%	0%	0%	7%	0%	0%	0%		-	-	-	-	-	-	0%	0%	0%	0%	0%	0%	0%	0%	0%	0%	0%	0%	20%	0%		17%	0%	
	Idiomatic	18%	21%	13%	0%	7%	12%	63%	23%		-	-	-	-	-	-	0%	24%	0%	0%	6%	0%	57%	8%	0%	0%	0%	0%	60%	0%		17%	0%	
	Syntactic/grammatical	9%	21%	7%	0%	14%	10%	25%	15%		-	-	-	-	-	-	0%	0%	0%	0%	6%	0%	43%	8%	0%	200%	0%	0%	200%	4%		17%	0%	
Spanish	Semantic	9%	47%	73%	50%	14%	19%	13%	8%		-	-	-	-	-	-	0%	33%	20%	100%	24%	20%	0%	5%	0%	200%	17%	0%	120%	7%		200%	0%	
	Cultural	0%	0%	0%	0%	7%	0%	0%	0%		-	-	-	-	-	-	0%	0%	0%	0%	0%	0%	0%	0%	0%	0%	0%	0%	0%	0%		17%	0%	
	Idiomatic	9%	5%	33%	100%	14%	7%	0%	0%		-	-	-	-	-	-	0%	29%	0%	100%	0%	0%	14%	14%	0%	200%	33%	20%	80%	0%		117%	0%	
	Syntactic/grammatical	0%	5%	0%	0%	0%	0%	0%	0%		-	-	-	-	-	-	0%	10%	0%	0%	0%	0%	29%	0%	0%	0%	0%	0%	0%	0%		17%	0%	
Swedish	Semantic	218%	32%	33%	200%	21%	10%	13%	8%		-	-	-	-	-	-	300%	52%	0%	300%	29%	40%	-	-	-	-	-	-	80%	24%		-	-	
	Cultural	0%	0%	0%	0%	0%	0%	0%	0%		-	-	-	-	-	-	0%	0%	0%	100%	0%	0%	-	-	-	-	-	-	0%	0%		-	-	
	Idiomatic	9%	0%	0%	0%	0%	0%	0%	0%		-	-	-	-	-	-	0%	0%	0%	100%	0%	0%	-	-	-	-	-	-	0%	0%		-	-	
	Syntactic/grammatical	73%	26%	7%	50%	14%	14%	63%	0%		-	-	-	-	-	-	0%	24%	0%	400%	6%	0%	-	-	-	-	-	-	140%	0%		-	-	

Note. In = instructions; I = items; R = response categories; ClinRO = clinician-reported outcome; PROM = patient-reported outcome; PerfO = performance-based outcome; - = already existing translated and linguistically validated questionnaires; GOSE = Glasgow Outcome Scale - Extended; instructions include introduction (1), commentary on the questions (9), and scoring (1); GOSE-Q = Glasgow Outcome Scale - Extended questionnaire version; instructions include introduction and header (1) and explanatory example for the item 9; different types of responses (dichotomous yes/no and polytomous item-related responses) result in 42 elements; GOAT = Galveston Orientation Amnesia Test (no response categories); GAD-7 = Generalized Anxiety Disorder 7 Items Questionnaire; PHQ-9 = Patient Health Questionnaire 9; PCL-5 = Posttraumatic Stress Disorder Checklist; RPQ = Rivermead Post-Concussion Symptoms questionnaire; QOLIBRI = Quality of Life after Brain Injury Scale; instructions include introduction to the two parts (2) and five subscales (5); QOLIBRI-OS = Quality of Life after Brain Injury – Overall Scale; RAVLT = Rey Auditory Verbal Learning Test; here, words are treated as items (5 x 15 = 45), (no response categories); instructions include introduction (1), explanations on the three trials (3), and the summary table for evaluation of the test result (1); TMT-A/B = Trail-Making Test A, B; in the TMT-A/B, letters and numbers are treated as items, (no response categories); instructions include introduction (1), explanation on the trial A (1) and trial B (1), trial B test (1), scoring (1), hands check (1); CANTAB = Cambridge Neuropsychological Test Automated Battery.

Interpretation: The table provides an overview of the differences between *the original English version* and the *first harmonization in the target languages* concerning instructions, items, and response categories on the semantic, cultural, idiomatic/pragmatic, and syntactic/grammatical level. The number of differences is expressed as a percentage (i.e., number of differences relative to the total number of respective text elements). The number varies from 0% (no differences at all) to 400% (multiple differences). Values above 100% indicate multiple modifications in one text element (e.g., four coded differences in one instruction are expressed as 400%). The same modification in the same text elements is considered once (e.g., the use of the polite pronoun form across 10 items in a questionnaire is counted as one modification in the items).

Comparisons between the first draft translations and the final target language versions of one ClinRO, its questionnaire version, a clinical amnesia test, PROMs, and PerfO instruments

		ClinRO, its questionnaire version, and a clinical rating									PROM*																		PerfO*					
		GOSE			GOSE-Q			GOAT			GAD-7			PHQ-9			PCL-5			RPQ			QOLIBRI			QOLIBRI-OS			RAVLT			TMT A/B		
Language	Type of differences (*column*) and no. of elements (*row*)	19 items			14 items			10 items			7 items			9+1 items			20+1 items			16 (+ 1 additional item)			37 items			6 items			Three versions of two word lists, 15 words each			numbers and letters		
		In	*I*	*R*	*In*	*I*	*R*	*In*	*I*	*R*	*In*	*I*	*R*	*In*	*I*	*R*	*In*	*I*	*R*	*In*	*I*	*R*	*In*	*I*	*R*	*In*	*I*	*R*	*In*	*I*	*R*	*In*	*I*	*R*
		11	19	15	2	14	42	8	13	0	1	7	5	1	10	5	1	21	5	1	17	5	7	37	5	1	6	5	5	45	0	6	38	0
Bosnian/ Croatian/ Serbian	Semantic	0%	26%	0%	0%	0%	0%	0%	85%		0%	0%	0%	-	-	-	100%	24%	20%	0%	0%	20%	0%	14%	0%	0%	33%	0%	0%	0%		0%	0%	
	Cultural	0%	5%	0%	0%	7%	0%	0%	0%		0%	0%	0%	-	-	-	0%	0%	0%	0%	0%	0%	0%	0%	0%	0%	0%	0%	0%	0%		0%	0%	
	Idiomatic	0%	0%	0%	0%	0%	0%	0%	0%		0%	0%	0%	-	-	-	0%	0%	0%	0%	0%	0%	0%	0%	0%	0%	0%	0%	0%	0%		0%	0%	
	Syntactic/grammatical	0%	5%	0%	0%	29%	0%	0%	69%		0%	0%	0%	-	-	-	0%	29%	0%	0%	6%	0%	0%	5%	0%	0%	0%	0%	0%	0%		0%	0%	
Danish	Semantic	-	-	-	100%	0%	2%	0%	15%		-	-	-	-	-	-	100%	33%	20%	-	-	-	-	-	-	-	-	-	0%	2%		-	-	
	Cultural	-	-	-	50%	0%	0%	0%	0%		-	-	-	-	-	-	100%	0%	0%	-	-	-	-	-	-	-	-	-	0%	0%		-	-	
	Idiomatic	-	-	-	0%	0%	0%	0%	8%		-	-	-	-	-	-	0%	0%	20%	-	-	-	-	-	-	-	-	-	0%	0%		-	-	
	Syntactic/grammatical	-	-	-	0%	0%	0%	13%	0%		-	-	-	-	-	-	0%	48%	20%	-	-	-	-	-	-	-	-	-	20%	2%		-	-	
Dutch	Semantic	-	-	-	0%	0%	0%	0%	0%		-	-	-	-	-	-	0%	0%	0%	0%	6%	0%	-	-	-	-	-	-	0%	4%		0%	0%	
	Cultural	-	-	-	0%	0%	0%	0%	0%		-	-	-	-	-	-	0%	0%	0%	0%	0%	0%	-	-	-	-	-	-	0%	0%		0%	0%	
	Idiomatic	-	-	-	0%	0%	0%	0%	0%		-	-	-	-	-	-	0%	0%	0%	0%	0%	0%	-	-	-	-	-	-	0%	0%		0%	0%	
	Syntactic/grammatical	-	-	-	0%	7%	2%	25%	15%		-	-	-	-	-	-	0%	14%	0%	0%	0%	0%	-	-	-	-	-	-	40%	0%		0%	0%	
Finnish	Semantic	-	-	-	350%	21%	7%	0%	8%		-	-	-	-	-	-	0%	0%	0%	400%	0%	20%	-	-	-	-	-	-	80%	0%		83%	0%	
	Cultural	-	-	-	50%	0%	0%	100%	8%		-	-	-	-	-	-	0%	0%	0%	100%	6%	0%	-	-	-	-	-	-	0%	0%		0%	0%	
	Idiomatic	-	-	-	0%	0%	0%	0%	0%		-	-	-	-	-	-	0%	0%	0%	0%	0%	0%	-	-	-	-	-	-	0%	0%		0%	0%	
	Syntactic/grammatical	-	-	-	50%	7%	0%	25%	0%		-	-	-	-	-	-	0%	5%	0%	100%	0%	20%	-	-	-	-	-	-	20%	0%		17%	0%	
French	Semantic	-	-	-	50%	7%	0%	0%	0%		-	-	-	-	-	-	100%	19%	0%	0%	0%	20%	-	-	-	-	-	-	0%	0%		0%	0%	
	Cultural	-	-	-	0%	7%	0%	0%	0%		-	-	-	-	-	-	0%	5%	0%	0%	0%	0%	-	-	-	-	-	-	0%	0%		0%	0%	
	Idiomatic	-	-	-	0%	0%	0%	13%	8%		-	-	-	-	-	-	0%	5%	0%	0%	0%	0%	-	-	-	-	-	-	20%	0%		0%	0%	
	Syntactic/grammatical	-	-	-	0%	0%	2%	38%	8%		-	-	-	-	-	-	0%	19%	0%	0%	12%	0%	-	-	-	-	-	-	40%	2%		0%	0%	
German	Semantic	-	-	-	550%	86%	62%	38%	62%		-	-	-	-	-	-	900%	129%	40%	-	-	-	-	-	-	-	-	-	800%	9%		0%	0%	
	Cultural	-	-	-	0%	0%	0%	0%	0%		-	-	-	-	-	-	0%	0%	0%	-	-	-	-	-	-	-	-	-	60%	0%		0%	0%	
	Idiomatic	-	-	-	0%	0%	0%	38%	38%		-	-	-	-	-	-	0%	0%	0%	-	-	-	-	-	-	-	-	-	0%	9%		0%	0%	
	Syntactic/grammatical	-	-	-	100%	29%	21%	0%	8%		-	-	-	-	-	-	0%	0%	0%	-	-	-	-	-	-	-	-	-	0%	2%		0%	0%	
Hebrew	Semantic	36%	16%	13%	50%	7%	2%	0%	8%		-	-	-	-	-	-	0%	19%	0%	0%	6%	0%	43%	14%	100%	0%	50%	0%	60%	7%		0%	0%	
	Cultural	0%	0%	0%	50%	0%	0%	0%	0%		-	-	-	-	-	-	0%	0%	0%	0%	0%	0%	0%	0%	0%	0%	0%	0%	0%	7%		0%	0%	
	Idiomatic	0%	0%	0%	0%	0%	0%	0%	0%		-	-	-	-	-	-	0%	19%	0%	0%	6%	20%	0%	0%	0%	0%	0%	0%	0%	4%		0%	0%	
	Syntactic/grammatical	9%	21%	7%	50%	14%	2%	0%	0%		-	-	-	-	-	-	100%	19%	20%	100%	6%	0%	0%	5%	0%	0%	0%	0%	40%	0%		0%	0%	
Hungarian	Semantic	18%	37%	7%	50%	0%	0%	50%	8%		-	-	-	-	-	-	0%	0%	20%	0%	0%	0%	0%	8%	0%	0%	0%	0%	40%	9%		67%	0%	
	Cultural	0%	0%	0%	0%	0%	0%	0%	0%		-	-	-	-	-	-	0%	0%	0%	0%	0%	0%	0%	0%	0%	0%	0%	0%	0%	0%		0%	0%	
	Idiomatic	0%	0%	0%	0%	0%	0%	0%	8%		-	-	-	-	-	-	0%	5%	0%	0%	0%	0%	0%	0%	0%	0%	0%	0%	40%	2%		17%	0%	
	Syntactic/grammatical	55%	42%	7%	0%	0%	2%	0%	23%		-	-	-	-	-	-	0%	0%	0%	300%	6%	0%	0%	5%	0%	0%	0%	0%	40%	2%		117%	0%	
Italian	Semantic	36%	21%	0%	0%	0%	0%	0%	0%		-	-	-	-	-	-	0%	33%	0%	0%	6%	0%	-	-	-	-	-	-	0%	0%		0%	0%	
	Cultural	0%	0%	0%	0%	0%	0%	0%	0%		-	-	-	-	-	-	0%	5%	0%	100%	6%	0%	-	-	-	-	-	-	40%	0%		17%	0%	
	Idiomatic	9%	0%	0%	0%	0%	0%	0%	0%		-	-	-	-	-	-	0%	0%	0%	0%	12%	0%	-	-	-	-	-	-	0%	0%		0%	0%	
	Syntactic/grammatical	0%	11%	0%	0%	0%	0%	0%	0%		-	-	-	-	-	-	100%	19%	60%	0%	0%	0%	-	-	-	-	-	-	80%	0%		0%	0%	
Latvian	Semantic	0%	0%	0%	0%	0%	0%	0%	0%		0%	0%	0%	0%	0%	0%	0%	0%	0%	0%	0%	0%	14%	3%	0%	0%	0%	0%	0%	0%		0%	0%	
	Cultural	0%	0%	0%	0%	0%	0%	0%	0%		100%	0%	0%	0%	0%	0%	0%	0%	0%	0%	6%	0%	14%	3%	0%	0%	17%	0%	0%	0%		0%	0%	
	Idiomatic	0%	0%	0%	0%	0%	0%	0%	0%		0%	0%	0%	0%	0%	0%	0%	0%	0%	0%	0%	0%	0%	3%	0%	0%	0%	0%	0%	0%		0%	0%	
	Syntactic/grammatical	9%	5%	0%	50%	7%	0%	50%	0%		0%	0%	29%	0%	20%	0%	100%	5%	0%	100%	0%	0%	57%	19%	0%	100%	0%	0%	20%	0%		17%	0%	
Lithuanian	Semantic	18%	21%	7%	50%	29%	2%	0%	8%		-	-	-	-	-	-	0%	0%	40%	400%	6%	0%	43%	3%	0%	0%	17%	0%	20%	0%		0%	0%	
	Cultural	9%	5%	0%	0%	0%	0%	0%	0%		-	-	-	-	-	-	0%	0%	0%	0%	0%	0%	0%	0%	0%	0%	0%	0%	0%	0%		0%	0%	
	Idiomatic	0%	0%	0%	0%	0%	0%	0%	0%		-	-	-	-	-	-	0%	10%	0%	0%	0%	0%	0%	0%	0%	0%	0%	0%	0%	0%		0%	0%	
	Syntactic/grammatical	9%	0%	7%	50%	14%	5%	0%	0%		-	-	-	-	-	-	0%	5%	0%	0%	0%	60%	0%	0%	0%	100%	17%	0%	80%	0%		67%	0%	
Norwegian	Semantic	-	-	-	0%	0%	2%	13%	8%		-	-	-	-	-	-	-	-	-	-	-	-	-	-	-	-	-	-	140%	2%		0%	0%	
	Cultural	-	-	-	0%	0%	0%	0%	0%		-	-	-	-	-	-	-	-	-	-	-	-	-	-	-	-	-	-	0%	0%		0%	0%	
	Idiomatic	-	-	-	0%	0%	0%	0%	0%		-	-	-	-	-	-	-	-	-	-	-	-	-	-	-	-	-	-	140%	4%		0%	0%	
	Syntactic/grammatical	-	-	-	0%	7%	0%	0%	0%		-	-	-	-	-	-	-	-	-	-	-	-	-	-	-	-	-	-	360%	2%		0%	0%	
Romanian	Semantic	9%	11%	0%	50%	0%	0%	13%	8%		-	-	-	-	-	-	0%	0%	0%	0%	0%	0%	0%	5%	0%	100%	0%	0%	0%	0%		0%	0%	
	Cultural	9%	5%	0%	0%	0%	0%	0%	0%		-	-	-	-	-	-	0%	0%	0%	0%	0%	0%	0%	0%	0%	100%	17%	0%	0%	0%		0%	0%	
	Idiomatic	0%	0%	0%	0%	0%	0%	0%	8%		-	-	-	-	-	-	0%	0%	0%	0%	0%	0%	0%	0%	0%	0%	0%	0%	0%	0%		0%	0%	
	Syntactic/grammatical	18%	5%	0%	0%	0%	0%	0%	0%		-	-	-	-	-	-	0%	0%	0%	0%	0%	0%	0%	0%	0%	100%	17%	0%	0%	0%		0%	0%	
Slovakian	Semantic	9%	16%	0%	200%	0%	12%	0%	0%		-	-	-	-	-	-	0%	14%	20%	500%	24%	0%	14%	5%	40%	0%	0%	40%	120%	4%		17%	0%	
	Cultural	0%	0%	0%	0%	7%	100%	0%	0%		-	-	-	-	-	-	0%	0%	0%	0%	0%	0%	0%	0%	0%	0%	0%	0%	33%	0%		17%	0%	
	Idiomatic	18%	0%	0%	100%	0%	7%	0%	31%		-	-	-	-	-	-	0%	10%	0%	0%	0%	20%	0%	5%	0%	100%	0%	0%	0%	0%		17%	0%	
	Syntactic/grammatical	18%	16%	0%	0%	21%	5%	0%	15%		-	-	-	-	-	-	300%	0%	0%	0%	0%	0%	0%	3%	100%	0%	0%	20%	120%	4%		33%	0%	
Spanish	Semantic	9%	16%	33%	100%	7%	17%	50%	38%		-	-	-	-	-	-	100%	10%	0%	0%	0%	20%	0%	27%	20%	100%	0%	0%	80%	7%		17%	0%	
	Cultural	0%	0%	13%	0%	0%	0%	0%	0%		-	-	-	-	-	-	0%	0%	0%	0%	18%	0%	0%	0%	0%	0%	0%	0%	0%	0%		0%	0%	
	Idiomatic	9%	5%	13%	0%	7%	2%	0%	8%		-	-	-	-	-	-	0%	0%	0%	0%	6%	0%	0%	0%	0%	200%	17%	20%	40%	7%		33%	0%	
	Syntactic/grammatical	0%	11%	20%	0%	14%	2%	0%	0%		-	-	-	-	-	-	0%	0%	0%	100%	0%	0%	0%	5%	0%	0%	0%	0%	100%	0%		0%	0%	
Swedish	Semantic	173%	58%	33%	50%	7%	5%	0%	8%		-	-	-	-	-	-	200%	114%	0%	300%	35%	40%	-	-	-	-	-	-	20%	9%		-	-	
	Cultural	0%	0%	0%	0%	0%	0%	0%	0%		-	-	-	-	-	-	0%	0%	0%	100%	0%	0%	-	-	-	-	-	-	0%	0%		-	-	
	Idiomatic	64%	0%	0%	0%	0%	0%	0%	0%		-	-	-	-	-	-	100%	10%	0%	300%	6%	0%	-	-	-	-	-	-	40%	2%		-	-	
	Syntactic/grammatical	82%	32%	13%	50%	7%	5%	0%	0%		-	-	-	-	-	-	100%	43%	0%	300%	18%	0%	-	-	-	-	-	-	20%	0%		-	-	

Note. In = instructions; I = items; R = response categories; ClinRO = clinician-reported outcome; PROM = patient-reported outcome; PerfO = performance-based outcome; - = already existing translated and linguistically validated questionnaires; GOSE = Glasgow Outcome Scale - Extended; instructions include introduction (1), commentary on the questions (9), and scoring (1); GOSE-Q = Glasgow Outcome Scale - Extended questionnaire version; instructions include introduction and header (1) and explanatory example for the item 9; different types of responses (dichotomous yes/no and polytomous item-related responses) result in 42 elements; GOAT = Galveston Orientation Amnesia Test (no response categories); GAD-7 = Generalized Anxiety Disorder 7 Items Questionnaire; PHQ-9 = Patient Health Questionnaire 9; PCL-5 = Posttraumatic Stress Disorder Checklist; RPQ = Rivermead Post-Concussion Symptoms questionnaire; QOLIBRI = Quality of Life after Brain Injury Scale; instructions include introduction to the two parts (2) and five subscales (5); QOLIBRI-OS = Quality of Life after Brain Injury – Overall Scale; RAVLT = Rey Auditory Verbal Learning Test; here, words are treated as items (5 x 15 = 45), (no response categories); instructions include introduction (1), explanations of the three trials (3), and the summary table for evaluation of the test result (1); TMT-A/B = Trail-Making Test A, B; in the TMT-A/B, letters and numbers are treated as items, (no response categories); instructions include introduction (1), explanation of the trial A (1) and trial B (1), trial B test (1), scoring (1), hands check (1); CANTAB = Cambridge Neuropsychological Test Automated Battery.

Interpretation: This table provides an overview of differences between the *first harmonization* and *the final version* in the target languages in instructions, items, and response categories at the semantic, cultural, idiomatic/pragmatic, and syntactic/grammatical level. The number of differences is expressed as a percentage (i.e., number of differences relative to the total number of respective text elements). The number varies from 0% (no differences at all) to 900% (multiple differences). Values above 100% indicate multiple modifications in one text element (e.g., nine coded differences in one instruction are expressed as 900%). The same modification in the same text elements is considered once (e.g., the use of the polite pronoun form across 10 items in a questionnaire is counted as one modification in the items).
